# Bioactive Monoterpenes and Polyketides from the Ascidian-Derived Fungus *Diaporthe* sp. SYSU-MS4722

**DOI:** 10.3390/md20090553

**Published:** 2022-08-29

**Authors:** Guifa Zhai, Senhua Chen, Hongjie Shen, Heng Guo, Minghua Jiang, Lan Liu

**Affiliations:** 1 School of Marine Sciences, Sun Yat-sen University, Zhuhai 519000, China; 2 School of Medicine, Shenzhen Campus, Sun Yat-Sen University, Shenzhen 518107, China; 3 Southern Laboratory of Ocean Science and Engineering (Guangdong, Zhuhai), Zhuhai 519000, China; 4 Pearl River Estuary Marine Ecosystem Research Station, Ministry of Education, Zhuhai 519082, China

**Keywords:** monoterpenes, α-pyrones, ascidian-derived fungus, *Diaporthe* sp., antioxidant and anti-inflammatory

## Abstract

There has been a tremendous increase in the rate of new terpenoids from marine-derived fungi being discovered, while new monoterpenes were rarely isolated from marine-derived fungi in the past two decades. Three new monoterpenes, diaporterpenes A–C (**1**–**3**), and one new α-pyrones, diaporpyrone A (**6**), along with nine known polyketides **4**, **5**, and **7**–**13** were isolated from the ascidian-derived fungus *Diaporthe* sp. SYSU-MS4722. Their planar structures were elucidated based on extensive spectroscopic analyses (1D and 2D NMR and HR-ESIMS). The absolute configurations of **1** and **3** were identified by an X-ray crystallographic diffraction experiment using Cu-Ka radiation, and those of compound **2** were assigned by calculating NMR chemical shifts and ECD spectra. It afforded an example of natural epimers with different physical properties, especially crystallization, due to the difference in intermolecular hydrogen bonding. Compounds **9**, **10**, and **13** showed moderate total antioxidant capacity (0.82 of **9**; 0.70 of **10**; 0.48 of **13**) with Trolox (total antioxidant capacity: 1.0) as a positive control, and compounds **5** and **7** showed anti-inflammatory activity with IC_50_ values of 35.4 and 40.8 µM, respectively (positive control indomethacin: IC_50_ = 35.8 µM).

## 1. Introduction

Marine fungal terpenoids are a kind of important metabolites with chemical structural diversity and pharmacological activity, such as cytotoxic, antibacterial, antifungal, antiviral, anti-inflammatory, and enzyme inhibitor activities [1,2]. In the past 20 years, there has been a tremendous increase in the rate of new terpenoids from marine-derived fungi being discovered [1,3,4], while monoterpenes were rarely isolated from marine-derived fungi. Up to now, only 12 new monoterpenes have been obtained from marine-derived fungi, including (1*S*,2*S*,3*S*,4*R*)-3-chloro-4-(2-hydroxypropan-2-yl)-1-methylcyclohexane-1,2-diol [5], pestalotiolactones C and D [6], penicipenes A and B [7], eutypellol B [8], 2-(2-hydroxy-4-methylcyclohex-3-enyl) propanoic acid [8], nectriapyrones C and D [9], (7*S*) and (7*R*)-1hydroxy-3-*p*-menthen-9-oic acids [10], 1-*O*-(α-d-mannopyranosyl) geraniol [11]. It is a challenging thing to discover new monoterpenes from marine-derived fungi.

The genus *Diaporthe* (asexual state: *Phomopsis*) comprises pathogenic, endophytic and saprobic species with both temperate and tropical distributions [12,13]. Chemical investigation of secondary metabolites and the discovery of novel bioactive natural products have been extensively researched because of their importance as plant pathogens [12]. For example, the mangrove-derived fungus *Diaporthe* sp. SCSIO 41011 produced six new highly oxygenated chloroazaphilone derivatives, isochromophilones A–F, with cytotoxicity [14]. Diaporisoindoles A–C were isolated from the mangrove endophytic fungus *Diaporthe* sp. SYSU-HQ3, which showed significant inhibitory activity against *Mycobacterium tuberculosis* protein tyrosine phosphatase B [15]. Dihydroisocoumarin, diaporone A, with antibacterial activity, was obtained from an endophytic plant fungus *Diaporthe* sp. [16].

**Figure 1:**
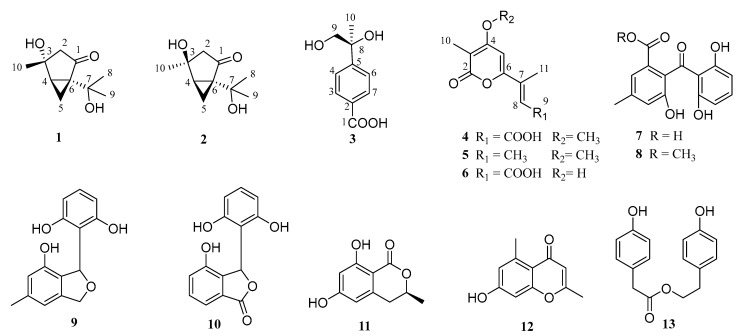
Chemical structures of compounds **1**–**13**.

Recently, our research group has focused on the secondary metabolites of ascidian-derived fungi isolated from the South China Sea [17,18,19,20]. Twenty mono- and dimeric xanthones with anti-glioma and anti-inflammatory activities have been obtained from the ascidian-derived fungus *Diaporthe* sp. SYSU-MS4722 [21]. Continuous chemical investigation of the EtOAc extract of marine fungus *Diaporthe* sp. SYSU-MS4722 led to the isolation of three new monoterpenes, diaporterpenes A–C (**1**–**3**), and one new α-pyrone, diaporpyrone A (**6**), along with nine known compounds **4**, **5**, and **7**–**13** (Figure 1) Herein, the details of the isolation, structural elucidation, and bioactivity of compounds **1**–**13** are reported.

## 2. Results and Discussion

The EtOAc extract of marine-derived fungus *Diaporthe* sp. SYSU-MS4722 was performed on repeated silica gel and Sephadex LH-20 column chromatography, followed by semipreparative HPLC to afford three new monoterpenes, diaporterpenes A–C (**1**–**3**), and one new α-pyrone, diaporpyrone A (**6**), along with nine known polyketides **4**, **5**, and **7**–**13**.

Diaporterpene A (**1**) was obtained as a colourless crystal with a melting point of 126–127 °C. The molecular formula was determined as C_10_H_16_O_3_ on the basis of the positive HR-ESIMS ions at *m*/*z* 185.1172 [M + H]^+^ (calculated for 185.1172, C_10_H_17_O_3_), implying three degrees of unsaturation. The IR spectrum (Supplementary Figure S8) of **1** revealed the presence of two hydroxy (3399 and 3325 cm^−1^) groups. The ^1^H NMR spectrum (Table 1) of **1** exhibited the signals for three tertiary methyls (*δ*_H_ 1.32, s; 1.40, s; 1.40, s), two methylenes (*δ*_H_ 1.97, dd, *J* = 0.6, 18.1 Hz; 2.38, dd, *J* = 2.1, 18.1 Hz; 0.96, dd, *J* = 4.6, 4.7 Hz; 1.48, ddd, *J* = 2.1, 5.0 8.1 Hz), and one methine (2.23, dd, *J* = 4.3, 8.1 Hz). The ^13^C NMR and DEPT data (Table 1) of 4 revealed the presence of 10 carbons belonging to one ketone carbonyl group (*δ*_C_ 214.3), two oxygen-bearing non-hydrogen carbon (*δ*_C_ 68.9, 73.9), one quaternary carbon (*δ*_C_ 47.7), one methine (*δ*_C_ 39.0), two methylenes (*δ*_C_ 16.1, 50.0), and three methyls (*δ*_C_ 25.4, 27.2, 27.7). These spectroscopic features suggested that 4 belongs to the family of monoterpenoids and is very similar to 2-thujanone, except for the presence of additional two oxygen-bearing non-hydrogen carbons (*δ*_C_ 68.9, 73.9) and the absence of two methines. 

**Figure 2:**
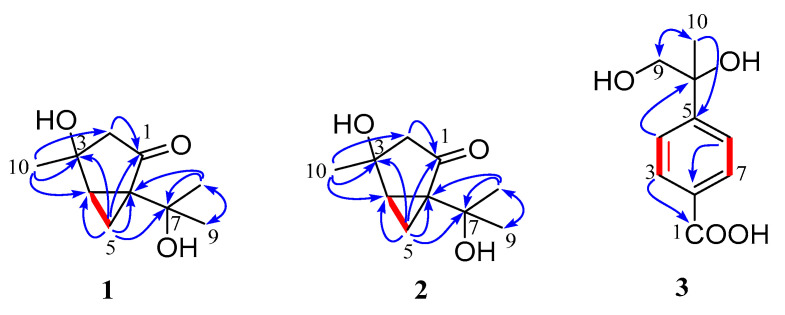
Key ^1^H-^1^H COSY (red line) and HMBC (blue arrow) correlations of compounds **1**–**3**.

**Figure 3:**
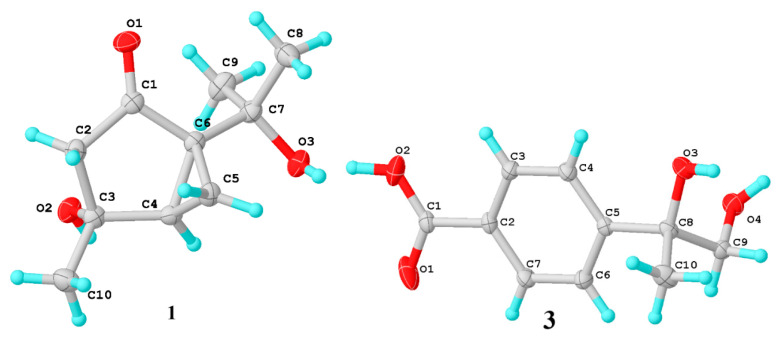
X-ray crystallographic analysis of **1** and **3**.

The planar structure was further assigned by 2D NMR spectra (^1^H-^1^H COSY, HSQC, HMBC) (Figure 2). The key HMBC correlations from H-2 to ketone C-1, from H-10 to C-2, C-3, C-4, from H-5 to C-2, C-3, C-4, C-6, C-7, and from H-8 and H-9 to C-6 and C-7, as well as ^1^H-^1^H COSY of H-4/H-5/H-6, indicated the presence of 1-isopropyl-4-methylbicyclo [3.1.0]hexan-2-one structure (2-thujanone). In the light of the chemical shifts (*δ*_C_ 73.9, C-3; 68.9, C-7), the remaining hydroxyl groups were located on C-3 and C-7, respectively. The absolute configuration of **1** was assigned as (3*R*,4*R*,6*S*) by a single-crystal X-ray diffraction experiment using Cu-Kα radiation (Figure 3) (Flack parameter = 0.09(19).

**Figure 4:**
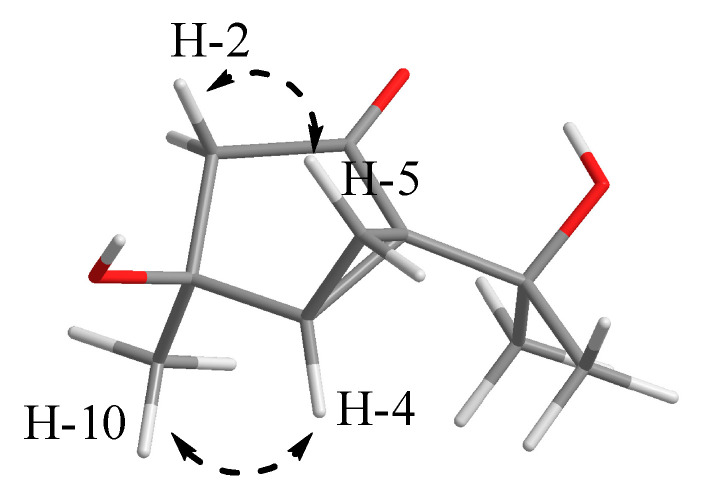
Key NOE (dash arrow) correlations of compound **2**.

**Figure 5:**
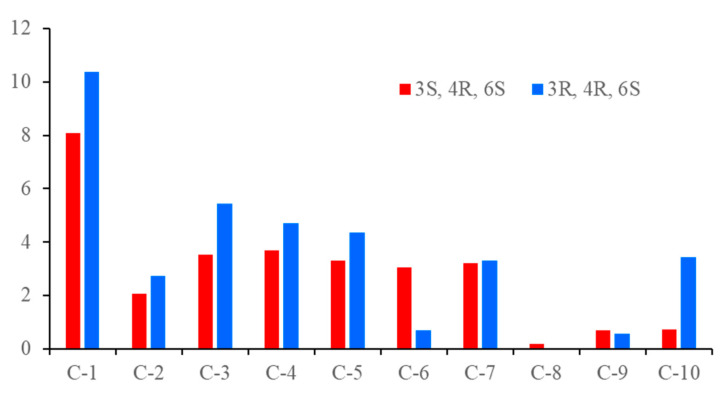
Values of the absolute deviation (|Δδ| = |δcalcd − δexptl|) for the possible structure of **2**.

**Figure 6:**
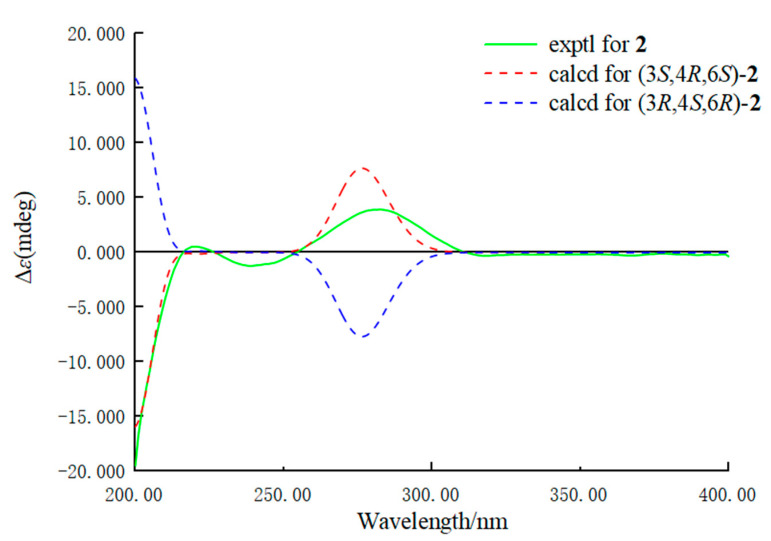
Experimental and predicted ECD spectra of **2** (in MeOH).

Diaporterpene B (**2**) was obtained as a colorless oil, and its molecular formula was identified as the same as **1** with C_10_H_16_O_3_ by the negative HRESIMS ions at *m*/*z* 183.1025 [M − H]^−^ (calculated for 183.1026, C_10_H_15_O_3_). Compound **2** shared the same planar structure as **1** and was further identified by 2D NMR spectra, including ^1^H-^1^H COSY, HSQC, and HMBC (Figure 2). The minor chemical shift variation of C-3 (*δ*_C_ 73.9 for **1**; *δ*_C_ 71.9 for **2**), C-5 (*δ*_C_ 16.1, *δ*_H_ 1.48 for **1**; *δ*_C_ 15.7, *δ*_H_ 1.52 for **2**), C-10 (*δ*_C_ 25.6, *δ*_H_ 1.40 for **1**; *δ*_C_ 27.2, *δ*_H_ 1.40 for **2**) were observed in same CD_3_OD solvent; this suggested that **2** should be a 3-epimer of **1**. The relative configuration of **2** was determined by analysis of the NOESY data’s difference between **1** and **2** (Figures S7 and S14). The additional presence of a NOESY correlation between H-10 and H-4 and the absence of NOESY correlation between H-10 and H-2α and H-5α in **2** indicated that H-10 and H-4 were the same facial sides (Figure 4). In order to determine the epimer relationship between **1** and **2**, the ^13^C NMR calculations of **1** (3-*epi*-**2**) and **2** at the B3LYP/6-311 + + G(2d,p) level were performed. The calculated NMR chemical shifts of **2** (3*S*, 4*R*, 6*S*) agreed better with the experimental data compared to those of 3-*epi*-**2** (3*R*, 4*R*, 6*S*) (Figure 5 and Figure S32). The DP4 probability analysis also revealed that **2** had 100% probability, while it had 0% probability for **1** (Figure S33). Finally, the absolute configuration was deduced by comparing experimental and calculated ECD spectra (Figure 6). The predicted ECD spectrum of (3*S*,4*R*,6*S*)-**2** was in good agreement with the experimental one. Thus, the absolute configuration of **2** was assigned as 3*S*,4*R*,6*S*. 

**Figure 7:**
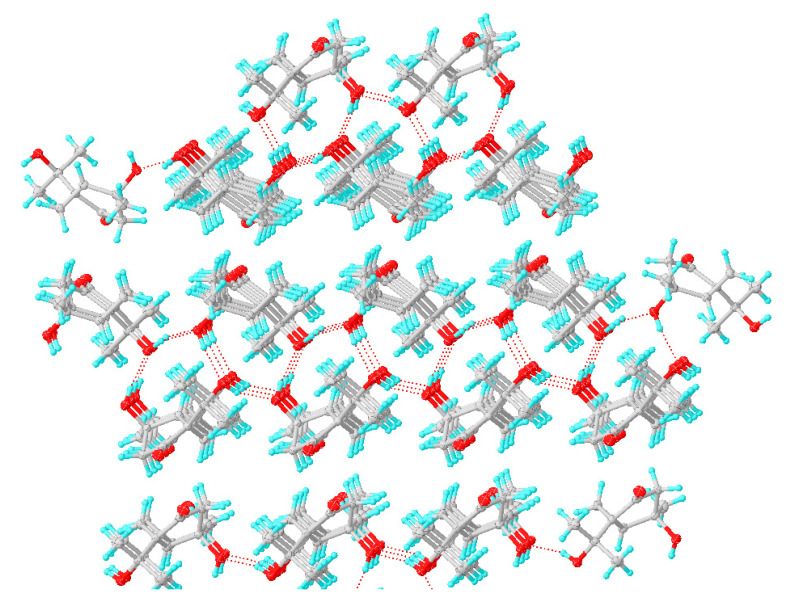
Single crystal molecular packing properties of **1**.

The monoterpenes, diaporterpenes A and B, were a pair of epimers with different physical properties, especially crystallization and solid-liquid states. Diaporterpene A was a solid state and had easy crystallization properties, while diaporterpene B was a liquid state and had difficult crystallization properties. Analysis of single crystal structure and molecular packing properties of **1** (Figure 7), intermolecular hydrogen bonds were observed. The 3-OH and 7-OH were the key functional groups for forming the intermolecular hydrogen bonds, while the O atom of the ketone does not make contributions. One molecule and four molecules around it together form five hydrogen bonding and look like a door handle. This is the main reason for the easy crystallization property for **1**. However, the absolute configuration of 3-OH in **2** was *S* with opposite orientation compared to **1**, which led to difficulty forming intermolecular hydrogen.

Diaporterpene C (**3**) was obtained as a colourless crystal. The molecular formula was determined as C_10_H_12_O_4_ on the basis of the negative HR-ESIMS ions at *m*/*z* 195.0666 [M − H]^−^ (calculated for 195.0663, C_10_H_11_O_4_), implying five degrees of unsaturation. The ^1^H NMR data (Table 1) revealed resonances for four para-substituted aromatic protons (*δ*_H_ 7.49 (2H, dd, *J* = 8.4 Hz); 7.88 (2H, d, *J* = 8.4 Hz)), one hydroxylmethyl (*δ*_H_ 3.52 (2H, q, *J* = 11.2 Hz)), and one tertiary methyl (*δ*_H_ 1.42, s). The ^13^C NMR and DEPT data (Table 1) of 5 revealed the presence of 10 carbons which were assigned with the help of an HSQC experiment. Except for seven sp^2^ hybridized carbons (*δ*_C_ 108.9, 130.4, 126.7, 152.9, 126.7, 130.4, 170.1) belonging to one benzoic acid, the remaining three sp^3^ hybridized carbons were sorted as methyl (*δ*_C_ 26.0), methylene (*δ*_C_ 71.6), and an oxygen-bearing non-hydrogen carbon (*δ*_C_ 75.7). The HMBC correlations from methyl H-10 and hydroxylmethyl H-9 to C-8 and C-5 of benzoic acid indicated a methyl, a hydroxylmethyl, and a hydroxyl were all connected to C-7 that was directly linked to C-5 of benzoic acid. Finally, the absolute configuration of **3** was assigned as *S* by a single-crystal X-ray diffraction experiment using Cu-Kα radiation (Flack parameter = −0.08(15) (Figure 3).

**Figure 8:**
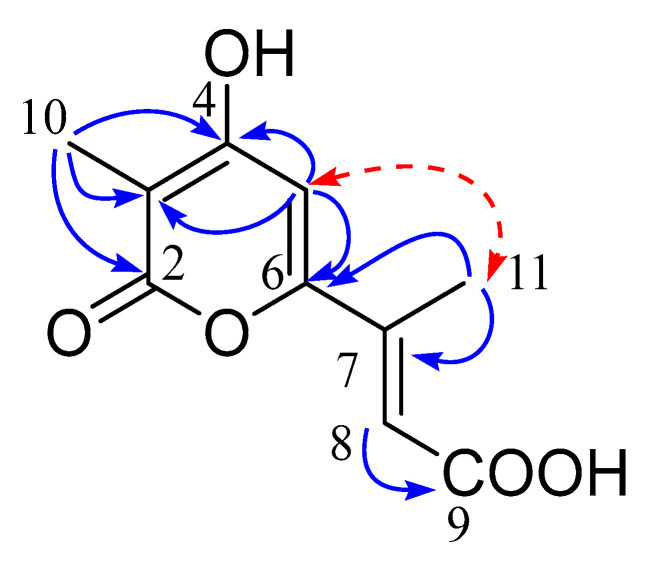
Key HMBC (blue arrow) and NOE (red dash arrow) correlations of compound **6**.

Diaporpyrone A (**6**) was obtained as a white powder, and its molecular formula C_10_H_10_O_5_ was established by the positive HRESIMS ion at *m*/*z* 209.0452 [M − H]^−^ (calculated for C_10_H_9_O_5_, 209.0455) showing six degrees of unsaturation. The ^1^H NMR spectrum displayed two olefinic protons at *δ*_H_ 6.45 (s) and 6.60 (d, *J* = 1.3 Hz) and two methyls (*δ*_H_ 1.88, s, and 2.88, s). The ^13^C NMR spectrum showed 10 carbons, including two conjugated carbonyls (*δ*_C_ 167.2, 169.8), six olefinic carbons (102.8, 103.3, 121.4, 142.7, 158.1, 166.8), and two sp^3^ carbons (8.7, 13.7). The key HMBC correlations of H-10 to C-2, C-3, and C-4, and H-5 to C-3, C-4, and C-6 suggested the presence of an α-pyrone moiety with a methyl substituent at C-3 (Figure 8). The HMBC correlations from H-11 to C-7 and H-8 to C-9 assigned a but-2-enoic acid moiety, which was connected to α-pyrone moiety via C-6. Key NOE correlation between H-5 with H-11 indicated that H-5 and H-11 were on the same side, while NOE correlation between H-8 with H-11 was not observed, suggesting that H-8 with H-11 were in the trans position. Hence, compound **6** was identified as shown in Figure 8. 

The known isolates were identified as acropyrone (**4**) [22], nectriapyrone (**5**) [23], monodictyphenone (**7**) [24], 2,2’,6’-trihydroxy-4-methyl-6-methoxy-acyl-diphenylmethanone (**8**) [25], pestacin (**9**) [26], 3-(2,6-dihydroxyphenyl)-4-hydroxy-6-methyl-isobenzofuran-1(3*H*)-one (**10**) [27], 3,4-dihydro-6,8-dihydroxy-3-methylisocoumarin (**11**) [28], 2,5-dimethyl-7-hydroxychromone (**12**) [25], and 4-hydroxyphenethyl 2-(4-hydroxyphenyl) acetate (**13**) [29] by comparison of their spectroscopic data with those reported in the literature. 

Phenolic compounds (including cinnamic acids, benzoic acids, flavonoids, proanthocyanidins, coumarins, stilbenes, lignans, and lignins) are the most widespread class of metabolites in nature and are proven to be effective antioxidants [30]. Compounds **1**–**13** were evaluated using the total antioxidant capacity assay kit with a rapid ABTS method. Only compounds **9**, **10**, and **13** showed moderate total antioxidant capacity (0.82 of **9**; 0.70 of **10**; 0.48 of **13**) with Trolox as a positive control. Anti-inflammatory activities were performed for compounds **1**–**13** with the inhibition of nitric oxide (NO) production in RAW264.7 cells activated by lipopolysaccharides, and compounds **5** and **7** showed anti-inflammatory activity with IC_50_ values 35.4 and 40.8 µM, respectively, with indomethacin (IC_50_ = 35.8 µM) as a positive control. All isolates were evaluated for anti-glioma activity using T98G human cell line with temozolomide as the positive control, and none of them showed significant cytotoxicity against T98G human cell line. 

## 3. Materials and Methods

### 3.1. General Experimental Procedures

Optical rotations were carried out on an MCP-200 polarimeter (Anton Paar, Graz, Austria) with MeOH as solvent at 25 °C. UV spectra were acquired on a Blue Star A spectrophotometer. IR data were performed on a Fourier transformation infrared spectrometer coupled with an EQUINOX 55 infrared microscope (Bruker, Fällanden, Switzerland). Here, 1D and 2D NMR spectra were tested on a Bruker Avance 400 MHz spectrometer (Bruker, Fällanden, Switzerland) using TMS as an internal standard. HRESIMS and ESIMS data were recorded on an LTQ-Orbitrap LC-MS spectrometer (Thermo Corporation, MA, USA) and an ACQUITY QDA (Waters Corporation, MA, USA), respectively. HPLC preparative separations were performed on a Shimadzu Essentia LC-16. The Welch-Ultimate XB-C18 column (250 × 21.2 mm, 5 μm, 12 nm, Welch Materials, Inc., Shanghai, China) was used for preparative HPLC. Semi-preparative HPLC separations were performed on ACE-5-C18-AR and ACE-5-CN-ES columns (250 × 10 mm, 5 μm, 12 nm, Advanced Chromatography Technologies Ltd., Guangzhou, China). The silica gel (200−300 mesh, Qingdao Marine Chemical Inc., Qingdao, China) and Sephadex LH-20 (Amersham Biosciences, Uppsala, Sweden) were subjected to column chromatography (CC).

### 3.2. Fungal Material

The detail of the fungus was described previously [21]. The molecular biological protocol, including DNA amplification and sequencing of the ITS region, was used for fungal identification. The sequence data of the fungal strain have been deposited at GenBank with accession no. OK623372. A BLAST search result suggested that the sequence was most similar (100%) to the sequence of *Diaporthe* sp. NFIF-2-6 (compared to MW202988.1). The strain was preserved at the School of Marine Sciences, Sun Yat-Sen University.

### 3.3. Extraction and Isolation

The strain *Diaporthe* sp. SYSU-MS4722 was grown on a solid rice medium in a 1000 mL culture flask containing 50g of rice and 50 mL of 3% artificial seawater after sterilization. A total of 120 flasks of fungal incubation were cultivated at room temperature for 30 days. The solid fermented substrate was extracted with MeOH four times to obtain a crude extract, then dissolved in H_2_O and continuously extracted four times with EtOAc solvent. The EtOAc extract (42 g) was subjected to a silica gel column eluting with linear gradient petroleum ether/EtOAc (from 8:2 to 0:1) to obtain six fractions (A–F). 

Fr.A was chromatographed on a Sephadex LH-20 column with MeOH/CH_2_Cl_2_ (50:50) to afford three fractions (Fr.A.1 to Fr.A.3) and compounds **9** and **13**. Fr.B was applied to the Sephadex LH-20 column with CH_2_Cl_2_/MeOH to provide four subfractions (Fr.B.1 to Fr.B.4). Fr.B.1 was further subjected to RP-HPLC (MeOH /H_2_O, 80:20 flow rate 2 mL/min, ACE-C18-PFP column 10 × 250 mm, 5 μm) to afford **11** (6.0 mg, *t*_R_ = 17.6 min). Fr.B.4 was applied to a silica gel column with CH_2_Cl_2_/MeOH (98:2) and then purified by RP-HPLC with MeOH/H_2_O (70:30) to give **12** (2 mg, *t*_R_ = 16.4 min). Fr.C was separated by a silica gel column eluting with CH_2_Cl_2_/MeOH (97:3) to afford five fractions (Fr.C.4.1 to Fr.C.4.5) and compound **8**. Fr.C.4.2 was further purified by a Sephadex LH-20 column with CH_2_Cl_2_/MeOH to give **4** (9.0 mg). Fr.C.4.3 was further purified by a silica gel column to give **5** (5.9 mg). Compounds **2** (20 mg, *t*_R_ = 13.4 min) were obtained from Fr.C.4.3 by silica gel CC (CH_2_Cl_2_/MeOH, 50:1, 30:1, 20:1, 10:1, v/v) with a final purification on a XB-C18 column (Welch column, MeCN/H_2_O, 14:86, 12 mL·min^−1^). Fr.C.4.5 was further purified by silica gel column to three fractions (Fr.C.4.5.1 to Fr.C.4.5.3), and Fr.C.4.5.2 was further purified by RP-HPLC (MeOH /H_2_O, 65:35 flow rate 2 mL/min, ACE-C18-PFP column 10 × 250 mm, 5 μm) to give **10** (9.0 mg, *t*_R_ = 10.8 min). Fr.C.4.5.3 was further purified by RP-HPLC (MeOH /H_2_O, 65:35 flow rate 2 mL/min, ACE-C18-PFP column 10 × 250 mm, 5 μm) to give **7** (9.0 mg). Fr.C.5 was subjected to silica gel chromatography to afford **6** (11 mg) and Fr.C.5.2, which was further purified by RP-HPLC (MeOH/H_2_O, 60:40 flow rate 2 mL/min, ACE-C18-PFP column 10 × 250 mm, 5 μm) to give **1** (5.0 mg, *t*_R_ =14.6 min). Fr.C.4.5 was further fractionated on a silica gel column and RP-HPLC to afford **3** (12 mg, *t*_R_ =19.5 min). 

Compound **1:** Colourless crystal; mp 126–127 °C; ${\left[\alpha \right]}_{D}^{20}$ −1.5 (*c* 1.00, MeOH); UV (MeOH) *λ*_max_ (log *ε*) 203 (3.48) nm; IR (neat) *ν*_max_ 3399, 3325, 2971, 2933, 1714, 1457, 1371, 1312, 1271, 1151, 1092, 928, 857 cm^−1^; HRESIMS *m/z* 185.1172 [M − H]^+^ (calculated for C_10_H_15_O_3_, 185.1172).

Compound **2:** brown oil; ${\left[\alpha \right]}_{D}^{20}$ −5.3 (*c* 1.00, MeOH); UV (MeOH) *λ*_max_ (log *ε*) 203 (3.48) nm; CD Δε (0.5 mg/mL, MeOH) *λ*_max_ (Δ*ε*) 218 (0.79), 240 (−1.15), 283 (3.98) nm; IR (neat) *ν*_max_ 3400, 2970, 2931, 1707, 1456, 1375, 1307, 1267, 1134, 1097, 1020, 947, 860 cm^−1^; ^1^H and ^13^C NMR data, see Table 2; HRESIMS *m/z* 183.10251 [M − H]^−^ (calculated for C_10_H_15_O_3_, 183.10267).

Compound **3:** Colourless crystal; mp 133−135 °C; UV (MeOH) *λ*_max_ (log ε) 232 (3.87) nm; IR (neat) *v*_max_ 3388, 1703, 1620, 1414, 1277, 1049 cm^−1^; HR-ESIMS *m*/*z* 195.0666 [M − H]^−^ (calculated for C_10_H_11_O_4_, 195.0663).

Compound **6:** Colourless powder; UV (MeOH) *λ*_max_ (log ε) 213 (4.05), 232 (3.89), 331 (3.93) nm; IR (neat) *v*_max_ 3215, 1668, 1585, 1416, 1254, 1076 cm^−1^; HR-ESIMS *m*/*z* 209.0452 [M − H]^−^ (calculated for C_10_H_9_O_5_, 209.0455).

### 3.4. X-ray Crystallographic Analysis

Compounds **1** and **3** were obtained as colorless crystals using the vapor diffusion method. The single crystal X-ray diffraction data were recorded on a Rigaku Oxford Diffraction with Cu-Kα radiation (λ = 1.54178A). The structures were solved by direct methods (SHELXS-97 and Olex2-1.2) and refined using full-matrix least-squares difference Fourier techniques. Crystallographic data for **1** and **3** have been deposited with the Cambridge Crystallographic Data Centre. Copies of the data can be obtained, free of charge, on application to the Director, CCDC, 12 Union Road, Cambridge CB2 1EZ, UK (fax: 44-(0)1223-336033, or e-mail: deposit@ccdc.cam.ac.uk).

Compound **1:** C_10_H_16_O_3_ (*Mr* = 184.23 g/mol), orthorhombic, space group P2_1_2_1_2_1_, *a* = 6.5845(2) Å, *b* = 7.8419(2) Å, *c* = 20.0925(6) Å, *α* = 90°, *β* = 90°, *γ* = 90°, *V* = 1037.48(5) Å^3^, *Z* = 4, *T* = 249.99(10) K, *µ*(Cu Kα) = 0.702 mm^−1^, *D*_calc_ = 1.179 g/cm^3^; 2039 reflections measured (*R*_int_ = 0.0387, *R*_sigma_ = 0.0581), which were used in all calculations. The final *R*_1_ was 0.0402 (*I* ≥ 2*u*(*I*)), and *wR*_2_ was 0.1031. The Flack parameter was 0.09(19). The goodness of fit on *F*^2^ was 1.053. CCDC 1997240.

Compound **3:** C_10_H_12_O_4_ (*Mr* = 196.20 g/mol), tetragonal, space group P4_3_2_1_2, *a* = 7.03910(10) Å, *b* = 7.03910(10) Å, *c* = 37.3747(6) Å, *α* = 90, *β* = 90, *γ* = 90, *V* = 1851.88(6) Å^3^, Z = 8, Crystal size 0.3 × 0.2 × 0.05 mm^3^, *µ*(Cu Kα) = 0.917 mm^−1^, *D*_calc_ = 1.407 g/cm^3^; 1874 reflections were measured (*R*_int_ = 0.0467, *R*_sigma_ = 0.0366), which were used in all calculations. The final *R*_1_ was 0.0440 (*I* ≥ 2*u*(*I*)), and *wR*_2_ was 0.1206. The Flack parameter was −0.08(15). The goodness of fit on *F*^2^ was 1.059. CCDC 1997237. 

### 3.5. Calculation of the ECD Spectra 

Merck molecular force field (MMFF) and DFT/TD-DFT calculations were performed with the Spartan’14 software package (Wavefunction Inc., Irvine, CA, USA) and the Gaussian 09 program (Gaussian, Inc., Wallingford, CT, USA), respectively [31]. MMFF conformational search generated low-energy conformers within a 10 kcal·mol^−1^ energy window. All the lowest energy conformers were subjected to DFT geometry optimizations at the *ω*B97X-D [32]/TZVP [33] level with the solvation model PCM for methanol. Frequency calculations were performed at the same level to confirm that each optimized conformer was at its true minimum and to estimate their relative thermal free energies (ΔG) at 298.15 K. Single-point energies were calculated at the M06-2X [34]/def2-TZVP [35]/SDM(MeOH) level of theory. The population of each conformer was calculated by Boltzmann distribution based on Gibbs free energy with the Shermo [36]. Finally, a free energy correction +1.89 kcal·mol^−1^ was applied to all free energies to consider the conversion from the gas phase (1 atm) to the liquid phase (1 M). Then, conformers with distributions higher than 1% were chosen for the TDDFT calculations at the PBE1PBE/TZVP [33] level in methanol. The ECD spectrum was generated by the program SpecDis [37] using a Gaussian band shape with 0.20 eV exponential half-width from dipole-length dipolar and rotational strengths. NMR calculations used the gauge-including atomic orbitals (GIAO) method at the mPW1PW91/6-311+G(d,p) level in MeOH simulated by the IEFPCM model. The TMS-corrected NMR chemical shift values were averaged according to Boltzmann distribution and fitted to the experimental values by linear regression. To confirm the conclusions of NMR calculations, DP4+ analysis was also performed. All calculations were performed by Tianhe-2 in National Super Computer Center in Guangzhou.

### 3.6. Anti-Glioma Activity

The assays for anti-glioma activity were evaluated as described previously [21]. The human glioma cell line, T98G, was purchased from the Cell Bank of the Chinese Academy of Sciences. Cells were cultured in Dulbecco’s modified Eagle’s medium (DMEM, Gibco, Carlsbad, CA, USA), containing 10% fetal bovine serum (FBS), 100 IU/mL penicillin, and 100 µg/mL streptomycin (all from Gibco, Carlsbad, CA, USA) in a cell incubator with 5% CO_2_ at 37 °C.

Cell proliferation was analyzed by MTT according to the manufacturer’s instructions. Briefly, T98G was digested and seeded at 1 × 10^3^ cells/well in 96-well plates and cultured in a 100 µL medium overnight. The cells were treated with tested compounds with gradient concentrations for 48 h. At each indicated time point, MTT solution (10 µL/well) was added and then incubated at 37 °C for 2 h. The optical density (OD) at 450 nm was recorded by a microplate reader (Multiskan GO, Thermo Scientific, Waltham, MA, USA). Each experiment was performed three times.

### 3.7. Anti-Inflammatory Activity

The assays for anti-inflammatory activity were performed as described previously [38]. RAW 264.7 cells were seeded in 96-well plates at a density of 5 × 10^5^ cells/ mL. After 12 h, the cells were treated with 1 µg/mL of LPS and tested samples, followed by additional incubation for 24 h at 37 °C. MTT stock solution (2 mg/mL) was added to wells for a total reaction volume of 100 µL. After 4 h incubation, the supernatants were aspirated. The formazan crystals in each well were dissolved in DMSO (100 µL), and the absorbance was measured with the wavelength of 490 nm by a microplate reader (Multiskan GO, Thermo Scientific). The data were expressed as mean percentages of the viable cells compared to the respective control. After pre-incubation of RAW 264.7 cells (1.5 × 10^5^ cells/mL) with 1 µg/mL LPS and samples at 37 °C for 24 h, the quantity of nitrite accumulated in the culture medium was measured as an indicator of NO production. Briefly, cell culture medium (50 µL) was added with Griess reagent (100 µL) and incubated at room temperature for 10 min. The absorbance was measured by a microplate reader (Multiskan GO, Thermo Scientific, Waltham, MA, USA) at 540 nm wavelength.

### 3.8. Total Antioxidant Capacity Assay

The assays for total antioxidant capacity were carried out as described previously [18]. A total antioxidant capacity assay kit with a rapid ABTS method (Beyotime Institute of Biotechnology, Shanghai, China) was used to evaluate the total antioxidant capacity based on the manufacturer’s instructions. Samples were incubated at 25 °C for 6 min and then were recorded at 414 nm using a multimode reader (Thermo Fisher Scientific, Waltham, MA, USA). 

## 4. Conclusions

It is a challenging thing to discover new monoterpenes from marine-derived fungi. Continuous chemical investigation of the EtOAc extract of marine fungus *Diaporthe* sp. SYSU-MS4722 afforded three new monoterpenes, diaporterpenes A–C (**1**–**3**), and one new α-pyrone, diaporpyrone A (**6**), along with nine known polyketides. Compounds **9**, **10**, and **13** showed moderate total antioxidant capacity (0.82 of **9**; 0.70 of **10**; 0.48 of **13**) with Trolox as a positive control, and compounds 5 and 7 showed anti-inflammatory activity with IC_50_ values of 35.4 and 40.8 µM, respectively. It afforded an example of natural epimers with different physical properties, especially crystallization, due to the difference in intermolecular hydrogen bonding.

## Figures and Tables

**Table 1:** ^1^H and ^13^C NMR spectroscopic data of **1**–**3** (CD_3_OD).

No.	1		2		3	
	*δ*_C_, type	*δ*_H_, mult (*J* in Hz)	*δ*_C_, type	*δ*_H_, mult (*J* in Hz)	*δ*_C_, type	*δ*_H_, mult (*J* in Hz)
1	214.3, C		211.9, C		170.1, C	
2	50.0, CH_2_	1.97, dd (0.6, 18.1) 2.38, dd (2.1, 18.1)	49.6, CH_2_	1.97, d (17.7) 2.44, d (17.7)	108.9, C	
3	73.9, C		71.9, C		130.4, CH	7.88, d (8.4)
4	39.0, CH	2.23, dd (4.3, 8.1)	38.1, CH	2.24, dd (4.4, 7.8)	126.7, CH	7.49, d (8.4)
5	16.1, CH_2_	0.96, dd (4.6, 4.7) 1.48, ddd (2.1, 5.0 8.1)	15.7, CH_2_	1.32, dd (4.6, 4.7) 1.52, ddd (1.6, 4.8, 7.6)	152.9, C	
6	47.7, C		50.3, C		126.7, CH	7.49, d (8.4)
7	68.9, C		68.5, C		130.4, CH	7.88, d (8.4)
8	27.2, CH_3_	1.32, s	29.2, CH_3_	1.28, s	75.7, C	
9	27.8, CH_3_	1.40, s	28.0, CH_3_	1.39, s	71.6, CH_2_	3.52, q (11.2)
10	25.6, CH_3_	1.40, s	27.2, CH_3_	1.41, s	26.0, CH_3_	1.42, s

**Table 2:** ^1^H (400 MHz) and ^13^C (100 MHz) NMR spectroscopic data of **6** (CD_3_OD).

No.	6	
	*δ*_C_, type	*δ*_H_, mult (*J* in Hz)
2	167.2, C	
3	102.8, C	
4	166.8, C	
5	103.3, CH	6.45, s
6	158.1, C	
7	142.7, C	
8	121.4, CH	6.60, d (1.3)
9	169.7, C	
10	8.7, CH_3_	1.88, s
11	13.5, CH_3_	2.30, s
12	OH	

## Supplementary Materials

The following are available online at https://www.mdpi.com/article/10.3390/md20090553/s1, Figure S1: The HRESIMS spectrum of compound **1**, Figures S2–S7: The 1 and 2D NMR (400MHz) spectra of compound **1** in CD_3_OD, Figure S8: The IR spectrum of compound **1**, Figure S9: The HRESIMS spectrum of compound **2**, Figure S10–S15: The 1 and 2D NMR (600MHz) spectra of compound **2** in CD_3_OD, Figure S16: The IR spectrum of compound **2**, Figure S17–S22: The 2D NMR (400MHz) spectra of compound **3** in CD_3_OD, Figure S23: The IR spectrum of compound **3**, Figure S24: The HRESIMS spectrum of compound **6**, Figure S25–S30: The 1 and 2D NMR (400MHz) spectra of compound **6** in CD_3_OD, Figure S31: The IR spectrum of compound **6**. Figure S32. Linear correlation between the experimental ^13^C NMR chemical shifts for **2**. Figure S33. The DP4 probability analysis. Table S1. Energy of all conformers of compound **2**. Figure S34. wB97XD/TZVP optimized low-energy conformers of compound **2**. Table S2. Cartesian coordinates of the low-energy reoptimized conformers of **2** calculated at PBE1PBE/TZVP level of theory in IEFPCM for methanol.
