# Supplementary material for: Bioactive Monoterpenes and Polyketides from the Ascidian-Derived Fungus Diaporthe sp. SYSU-MS4722
Source: Mar Drugs. 2022 Aug 29;20(9):553. doi: 10.3390/md20090553 (PMC9504586; doi:10.3390/md20090553)
Supplement: Supplementary file 1 [file marinedrugs-20-00553-s001.zip › marinedrugs-1887664-supplementary.pdf]

Supporting Information

Bioactive monoterpenes and polyketides from the ascidian-derived fungus *Diaporthe* sp. SYSU-MS4722

Guifa Zhai[1,2], Senhua Chen [1,3*], Hongjie Shen [1,3], Heng Guo [1], Minghua Jiang [1,3], and Lan Liu [1,3,4*]

[1] School of Marine Sciences, Sun Yat-sen University, Zhuhai 519000, China; zhaigf@mail.sysu.edu.cn; shenhj5@mail2.sysu.edu.cn; hengeguo163@163.com; jiangmh23@mail2.sysu.edu.cn;
[2] School of Medicine, Shenzhen Campus of Sun Yat-Sen University, Shenzhen 518107, China
[3] Southern Laboratory of Ocean Science and Engineering (Guangdong, Zhuhai), Zhuhai 519000, China
[4] Pearl River Estuary Marine Ecosystem Research Station, Ministry of Education, Zhuhai 519082, China.
* Correspondence: chensenh@mail.sysu.edu.cn; cesllan@mail.sysu.edu.cn; Tel.:020-84725459.

Figure S1. The HRESIMS spectrum of compound **1**....... 3

Figure S2. The ^{1}H NMR (400MHz) spectrum of compound **1** in CD_3OD....... 3

Figure S3. The ^{13}C NMR (100MHz) spectrum of compound **1** in CD_3OD....... 4

Figure S4. The HSQC spectrum of compound **1** in CD_3OD....... 4

Figure S5. The ^{1}H-^{1}H COSY spectrum of compound **1** in CD_3OD....... 5

Figure S6. The HMBC spectrum of compound **1** in CD_3OD....... 5

Figure S7. The NOESY spectrum of compound **1** in CD_3OD....... 6

Figure S8. IR spectrum of compound **1.**....... 6

Figure S9. The HRESIMS spectrum of compound **2**....... 7

Figure S10. The ^{1}H NMR (600MHz) spectrum of compound **2** in CD_3OD....... 7

Figure S11. The ^{13}C NMR (150MHz) spectrum of compound **2** in CD_3OD....... 8

Figure S12. The HSQC spectrum of compound **2** in CD_3OD....... 8

Figure S13. The ^{1}H-^{1}H COSY spectrum of compound **2** in CD_3OD....... 9

Figure S14. The HMBC spectrum of compound **2** in CD_3OD....... 9

Figure S15. The NOESY spectrum of compound **2** in CD_3OD....... 10

Figure S16. IR spectrum of compound **2** in CD_3OD....... 10

Figure S17. The HRESIMS spectrum of compound **3**....... 11

Figure S18. The ^{1}H NMR (400MHz) spectrum of compound **3** in CD_3OD....... 11

Figure S19. The ^{13}C NMR (100MHz) spectrum of compound **3** in CD_3OD....... 12

Figure S20. The HSQC spectrum of compound **3** in CD_3OD. 12

Figure S21. The ^{1}H-^{1}H COSY spectrum of compound **3** in CD_3OD. 13

Figure S22. The HMBC spectrum of compound **3** in CD_3OD. 13

Figure S23. IR spectrum of compound **3**. 14

Figure S24. The HRESIMS spectrum of compound **6**. 14

Figure S25. The ^{1}H NMR (400MHz) spectrum of compound **6** in CD_3OD. 15

Figure S26. The ^{13}C NMR (100MHz) spectrum of compound **6** in CD_3OD. 15

Figure S27. The HSQC spectrum of compound **6** in CD_3OD. 16

Figure S28. The ^{1}H-^{1}H COSY spectrum of compound **6** in CD_3OD. 16

Figure S29. The HMBC spectrum of compound **6** in CD_3OD. 17

Figure S30. The NOESY spectrum of compound **6** in CD_3OD. 17

Figure S31. IR spectrum of compound **6.** 18

Figure S32. Linear correlation between the experimental ^{13}C NMR chemical shifts for **2**. 18

Figure S33. The DP4 probability analysis 19

Table S1. Energy of all conformers of compound **2**. 19

Figure S34. wB97XD/TZVP optimized low-energy conformers of compound **2**. 21

Table S2. Cartesian coordinates of the low-energy reoptimized conformers of **2** calculated at PBE1PBE/TZVP level of theory in IEFPCM for methanol. 21

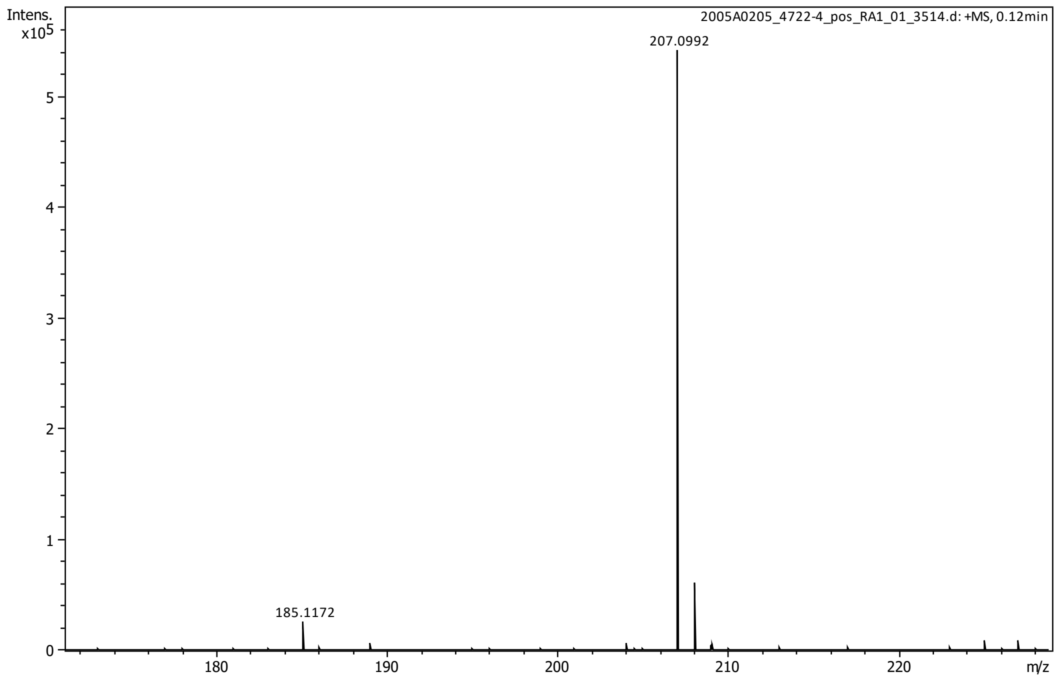

Meas. m/z	Ion Formula	m/z	z	err [ppm]	err [mDa]	mSigma	rdb	e⁻ Conf	N-Rule
185.1172	C10H17O3	185.1172	1+	0	0	8.7	3	even	ok
207.0992	C10H16NaO3	207.0992	1+	-0.1	0	1.7	3	even	ok

Figure S1. The HRESIMS spectrum of compound **1**.

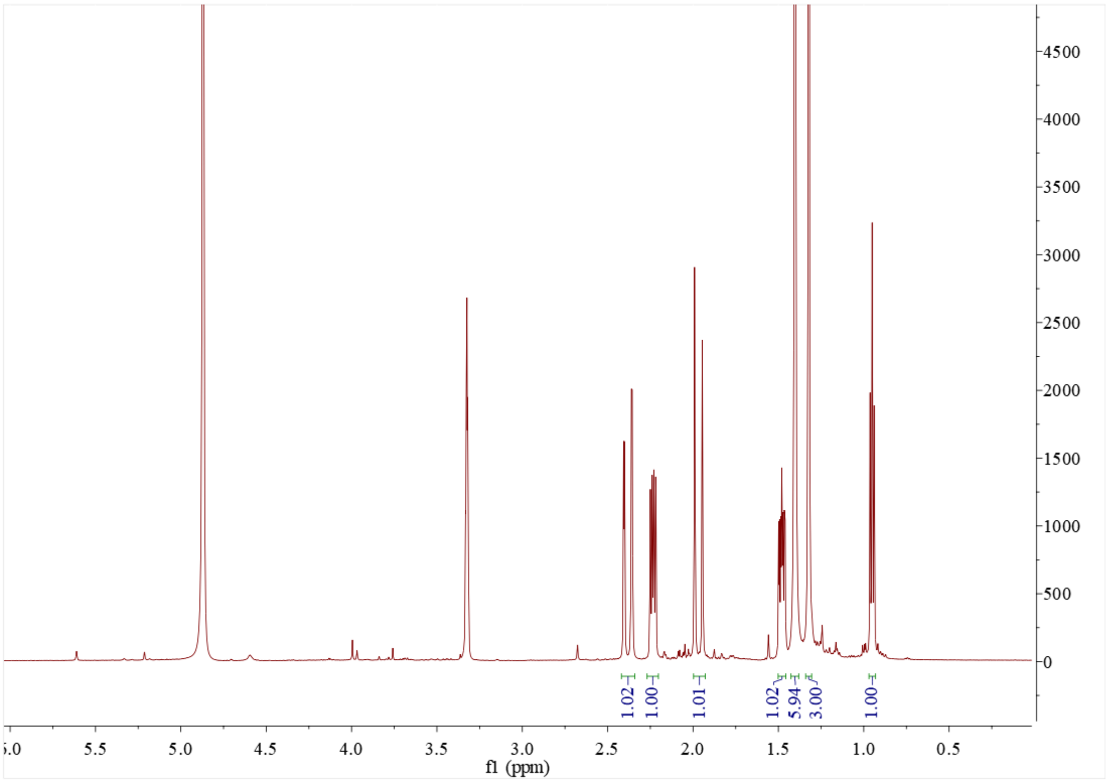

Figure S2. The ^{1}H NMR (400MHz) spectrum of compound **1** in CD_3OD.

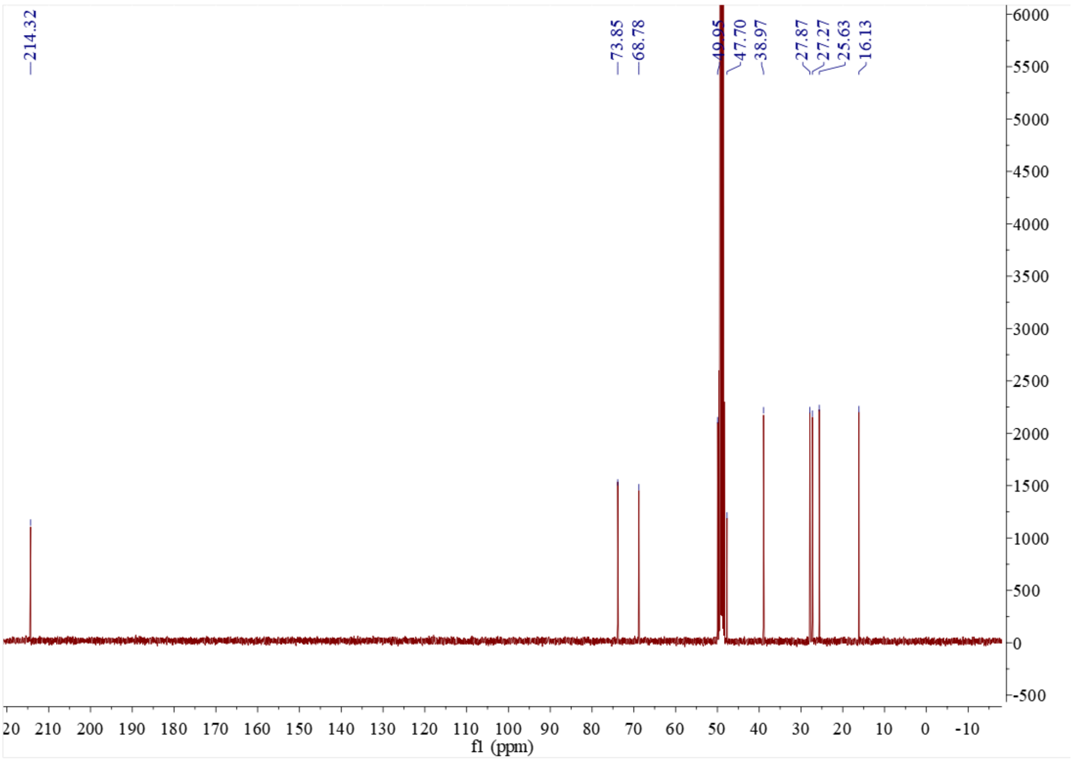

Figure S3. The ^{13}C NMR (100MHz) spectrum of compound **1** in CD_3OD.

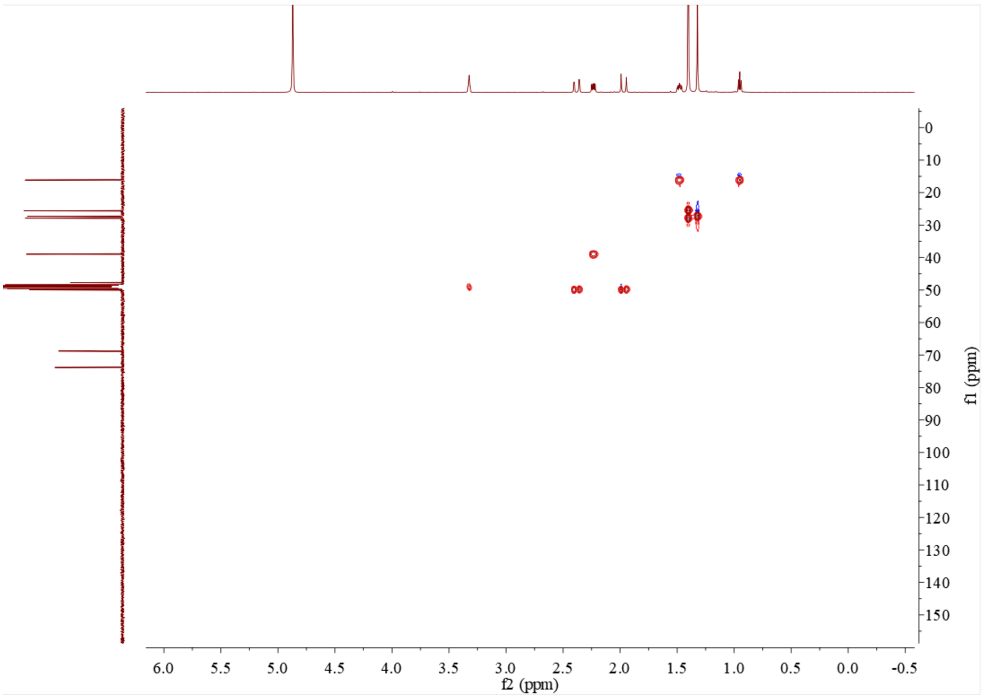

Figure S4. The HSQC spectrum of compound **1** in CD_3OD.

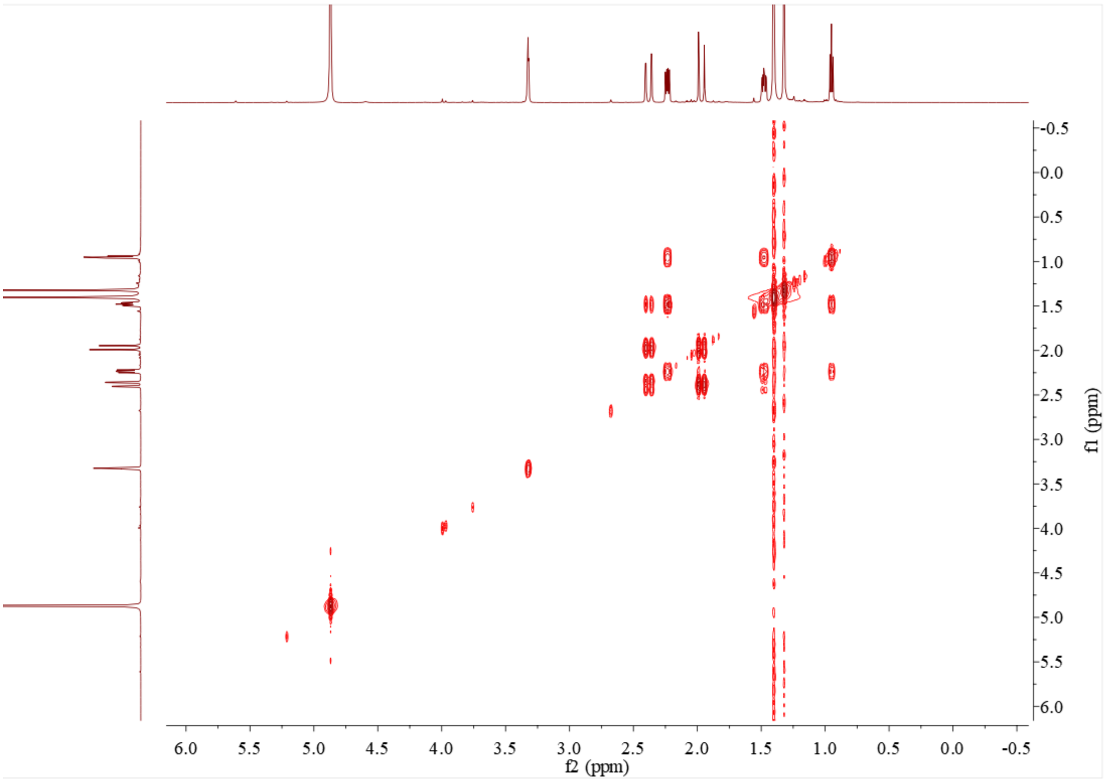

Figure S5. The ^{1}H-^{1}H COSY spectrum of compound **1** in CD_3OD.

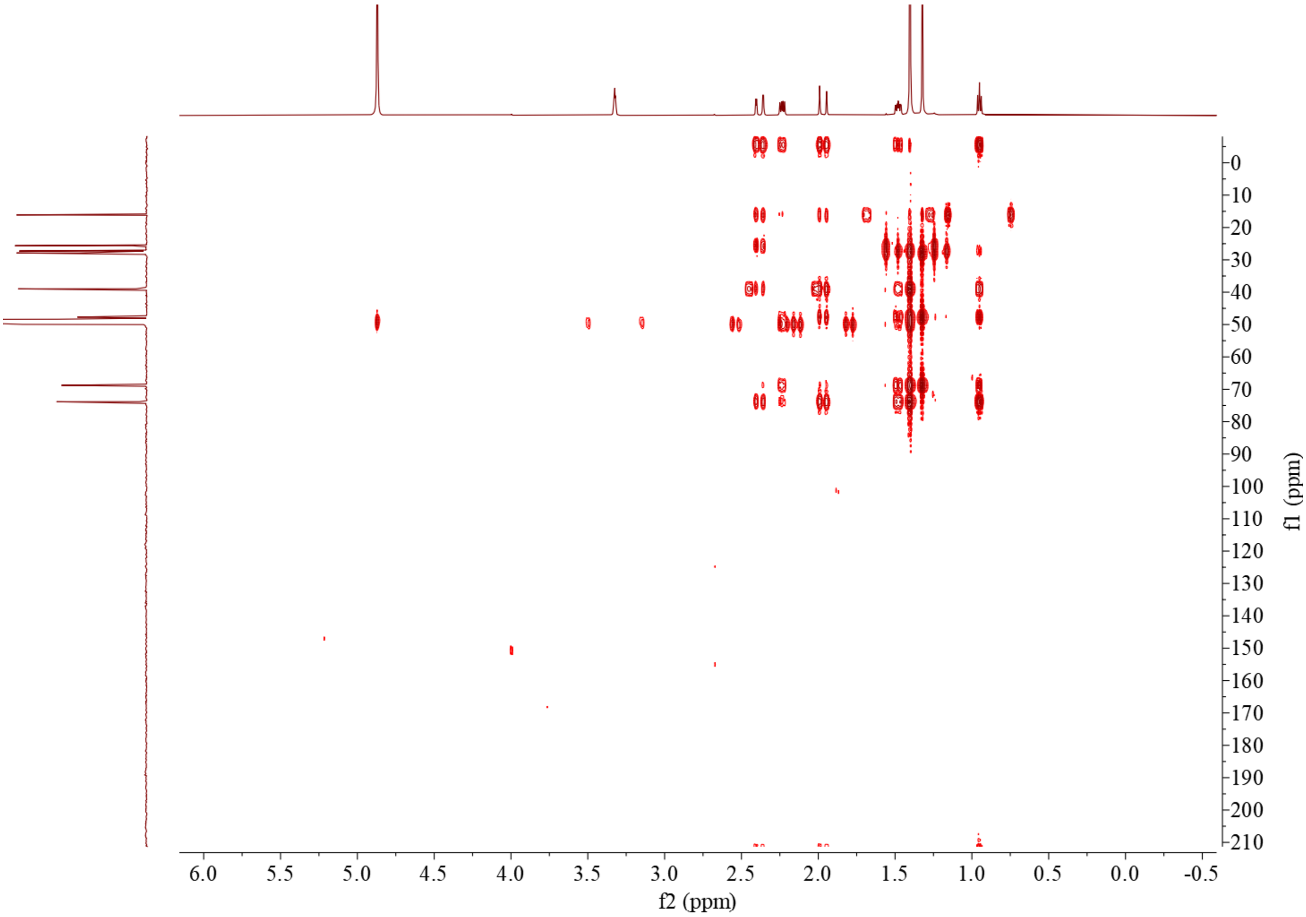

Figure S6. The HMBC spectrum of compound **1** in CD_3OD.

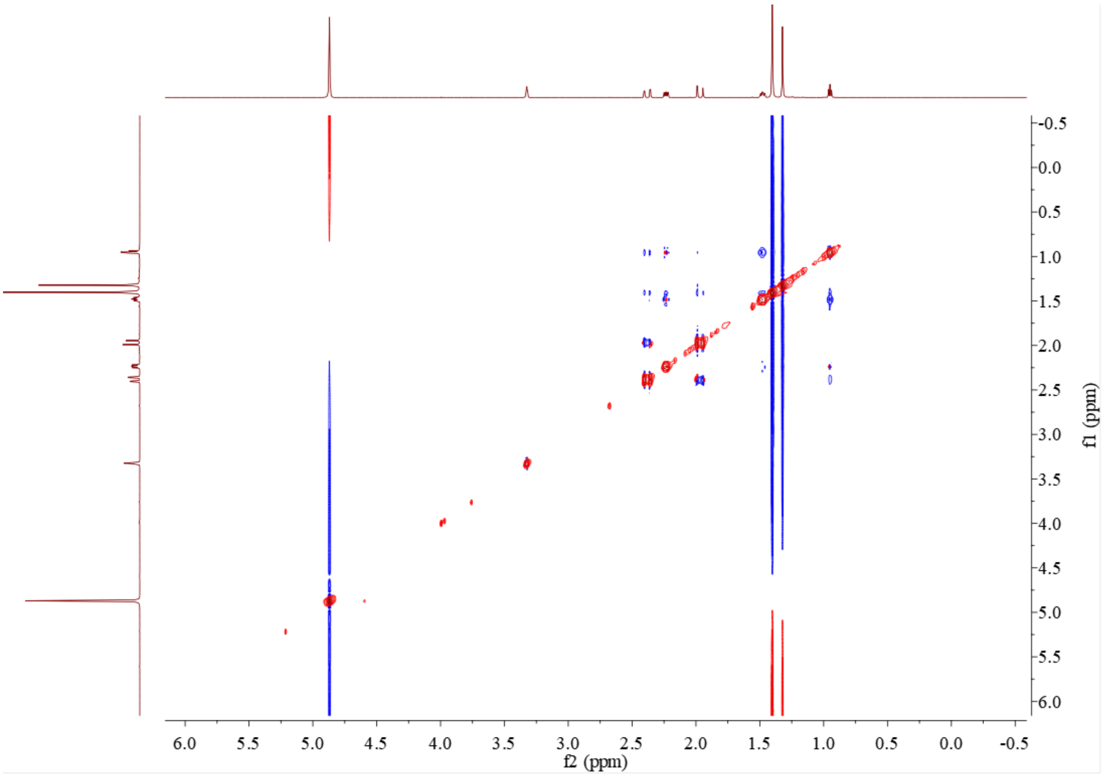

Figure S7. The NOESY spectrum of compound **1** in CD_3OD.

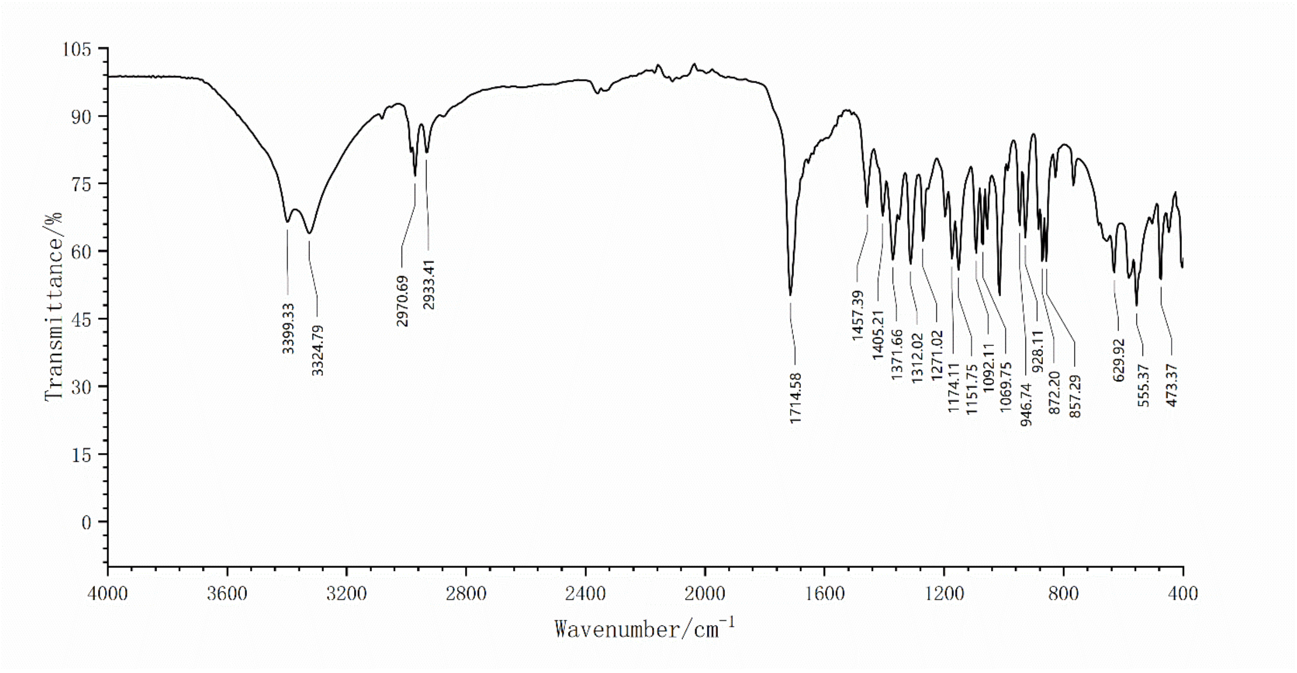

Figure S8. IR spectrum of compound **1.**

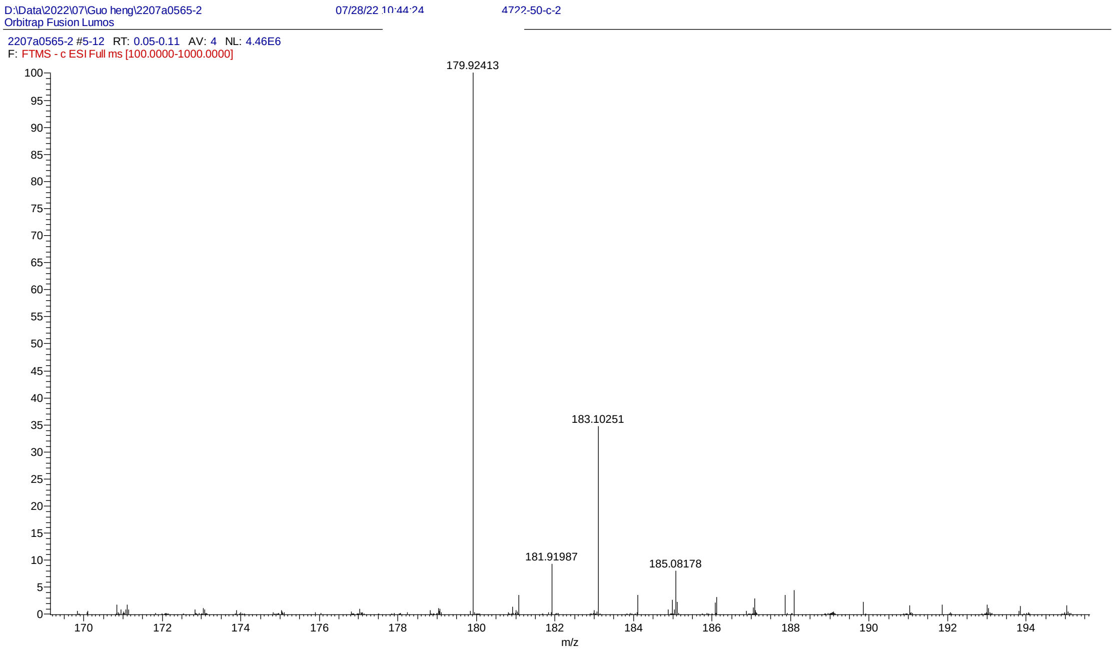

SPECTRUM - simulation :

m/z	Theo. Mass	Delta (ppm)	RDB equiv.	Composition
183.10251	183.10267	-0.86	3.5	C10 H15 O3

Figure S9. The HRESIMS spectrum of compound **2**.

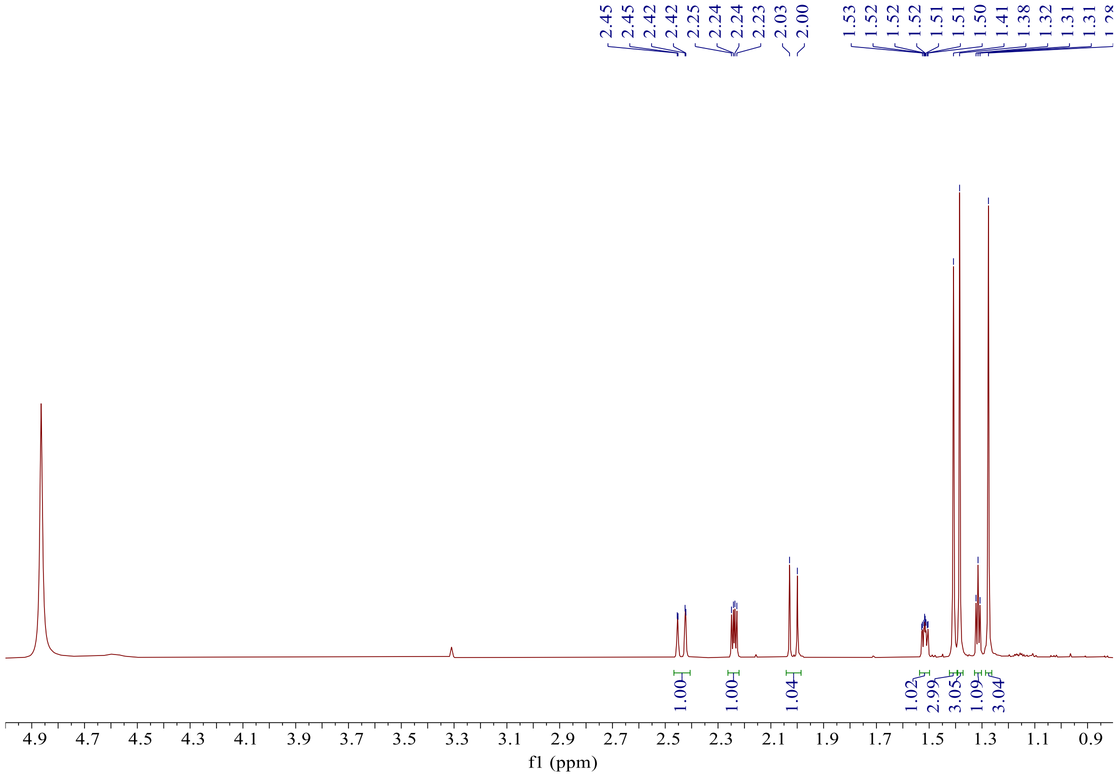

Figure S10. The ^{1}H NMR (600MHz) spectrum of compound **2** in CD_3OD.

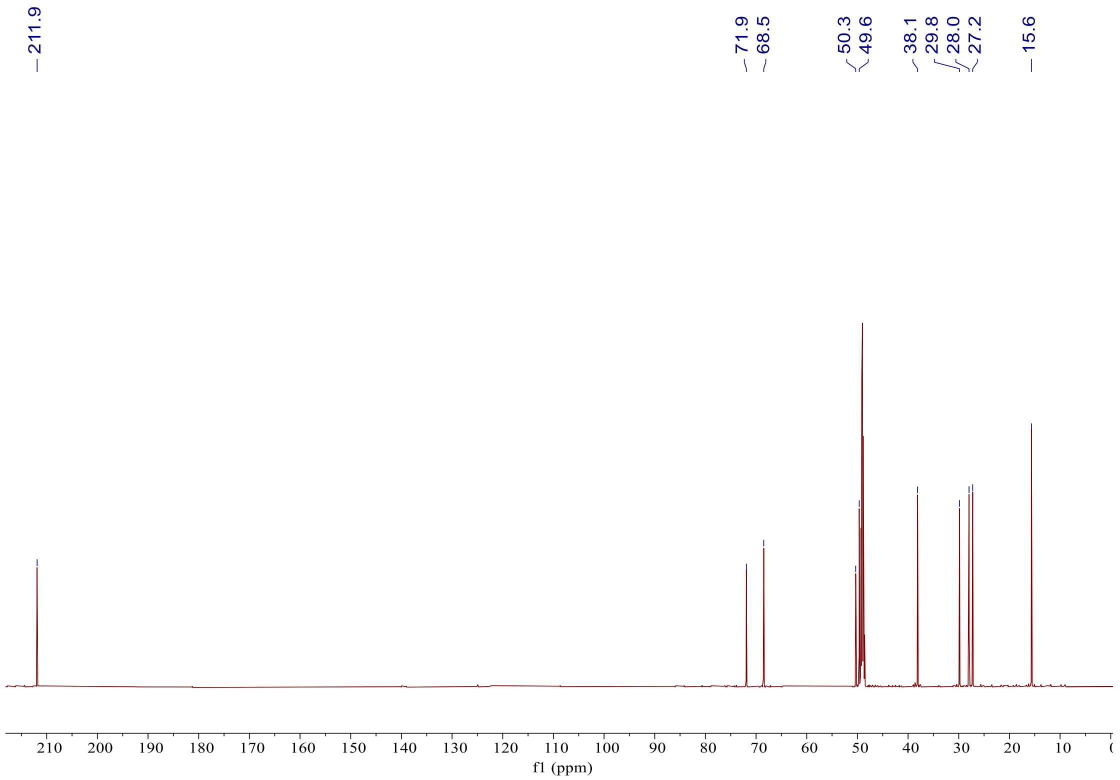

Figure S11. The ^{13}C NMR (150MHz) spectrum of compound **2** in CD_3OD.

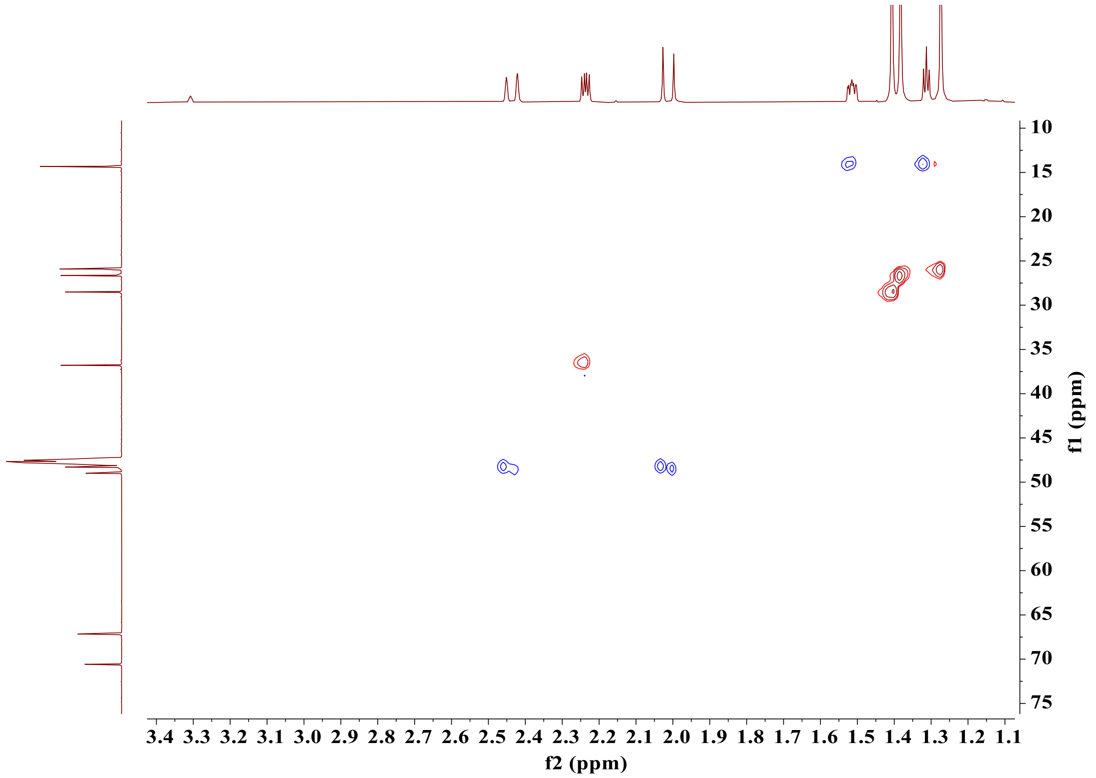

Figure S12. The HSQC spectrum of compound **2** in CD_3OD.

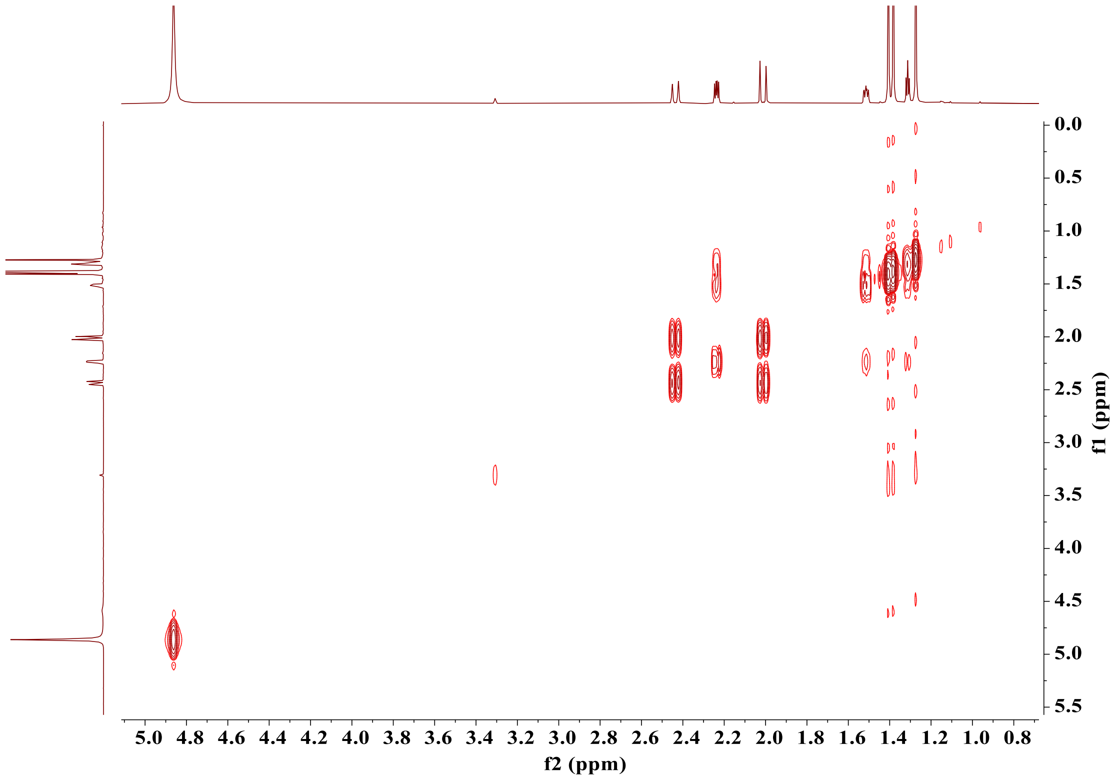

Figure S13. The ${}^{1}H$-${}^{1}H$ COSY spectrum of compound **2** in CD_3OD.

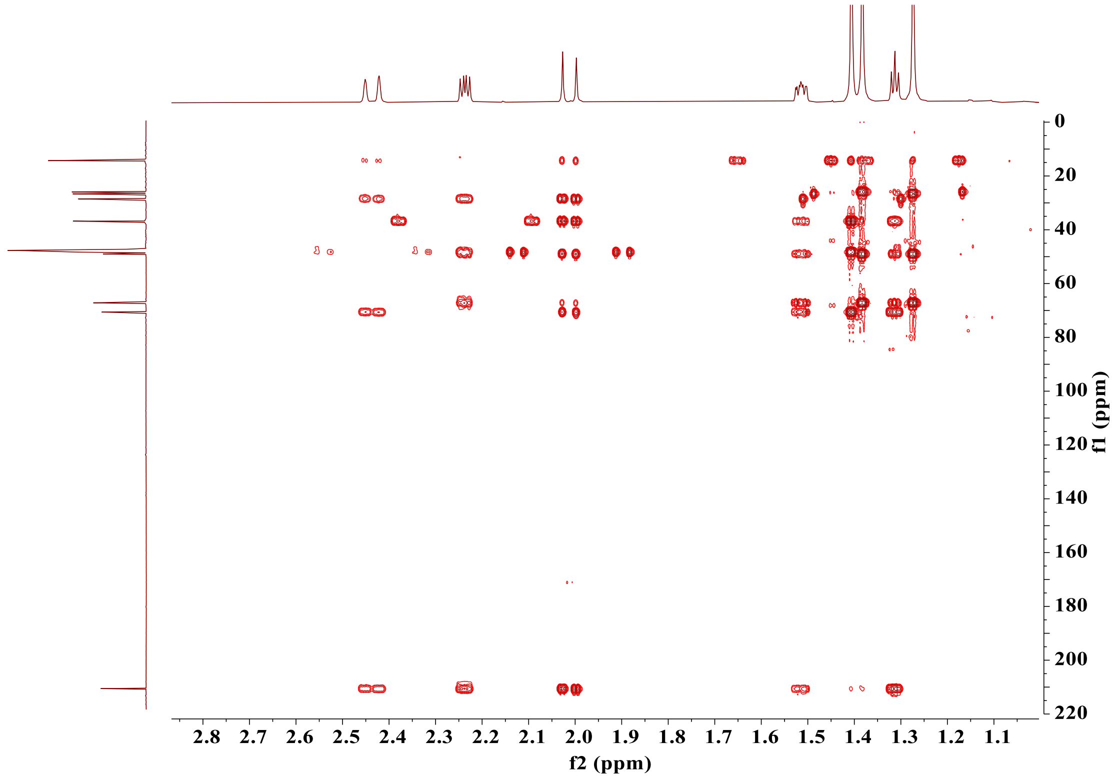

Figure S14. The HMBC spectrum of compound **2** in CD_3OD.

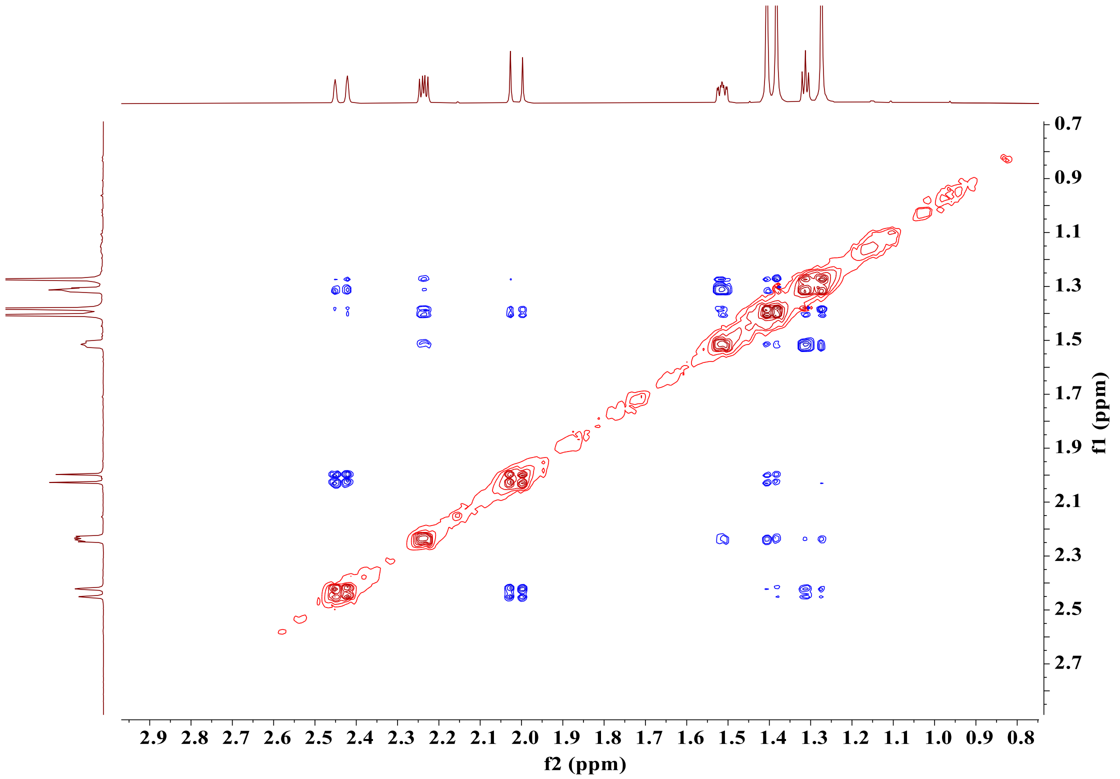

Figure S15. The NOESY spectrum of compound **2** in CD_3OD.

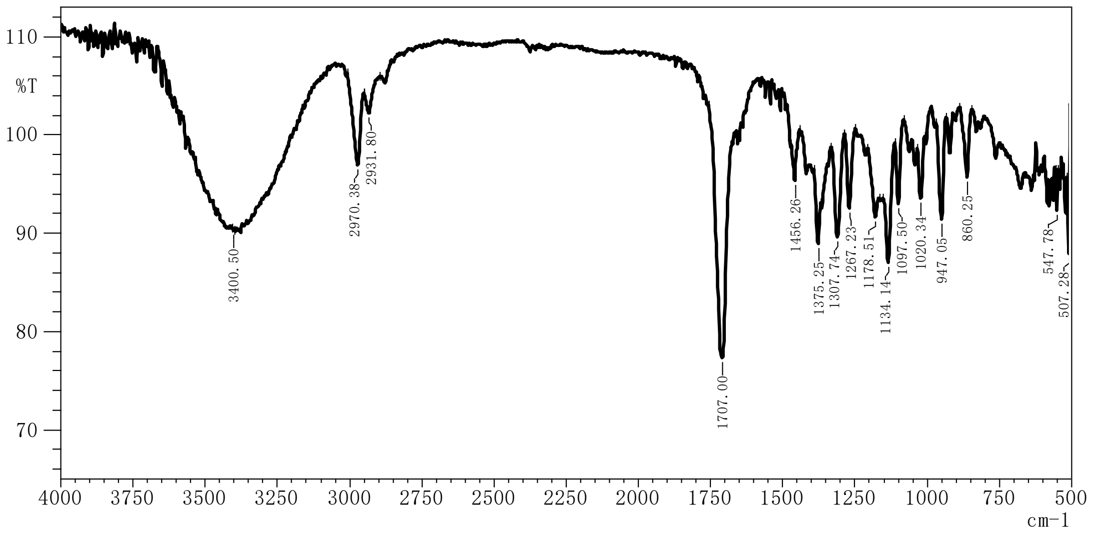

Figure S16. IR spectrum of compound **2** in CD_3OD.

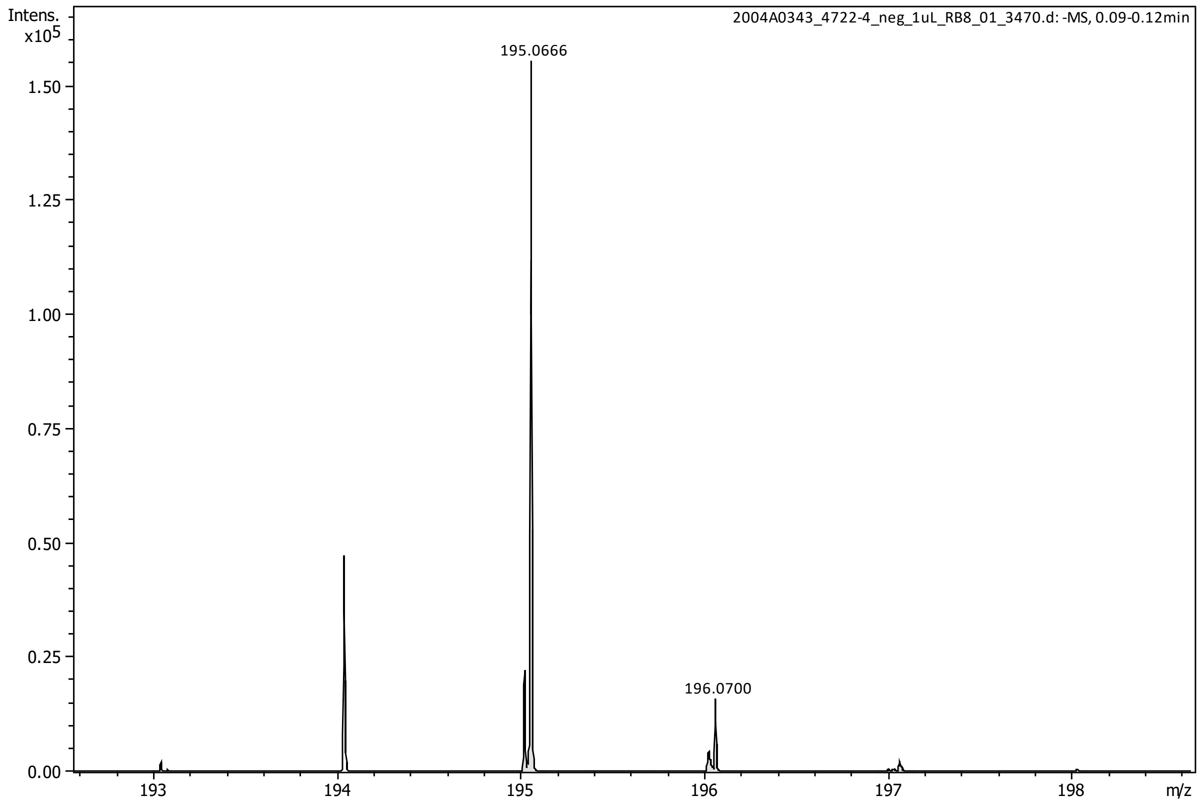

Meas. m/z	Ion Formula	m/z	z	err [ppm]	err [mDa]	mSigma	rdb	e⁻ Conf	N-Rule
195.0666	C10H11O4	195.0663	1-	-1.8	-0.3	4.4	5	even	ok

Figure S17. The HRESIMS spectrum of compound **3**.

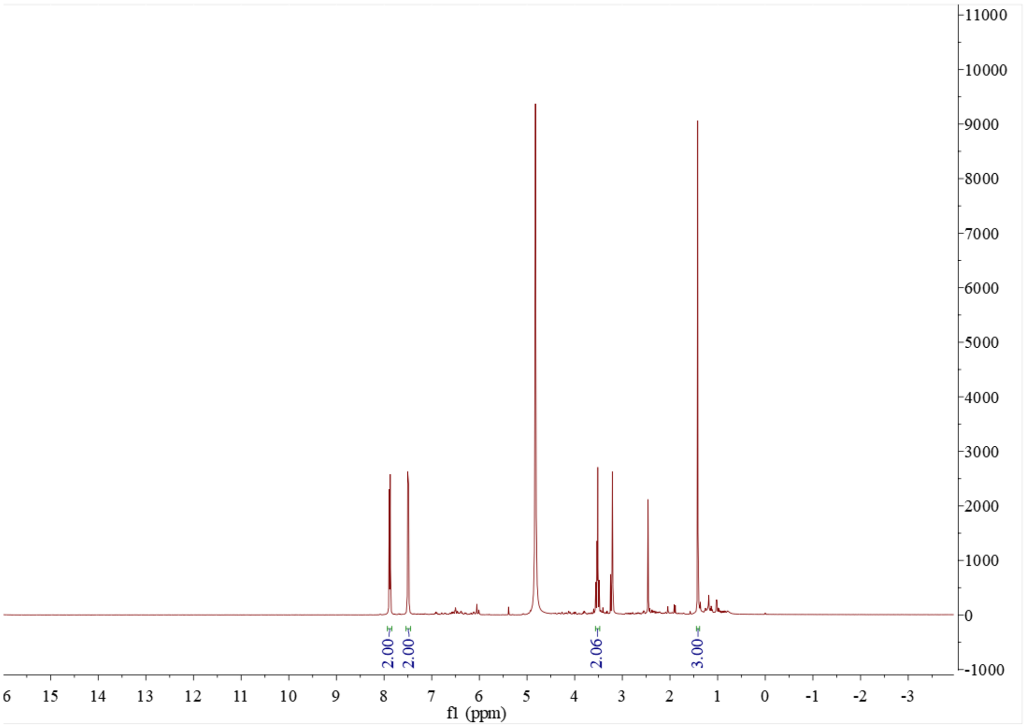

Figure S18. The ^{1}H NMR (400MHz) spectrum of compound **3** in CD_3OD.

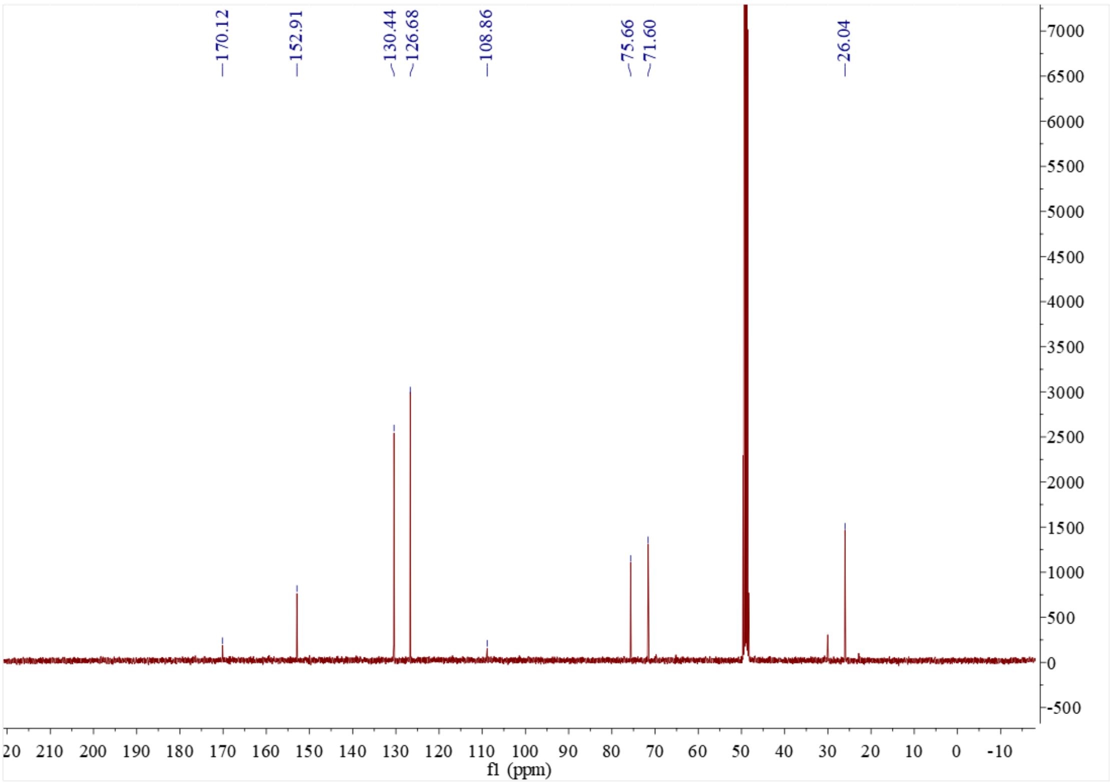

Figure S19. The ^{13}C NMR (100MHz) spectrum of compound **3** in CD_3OD.

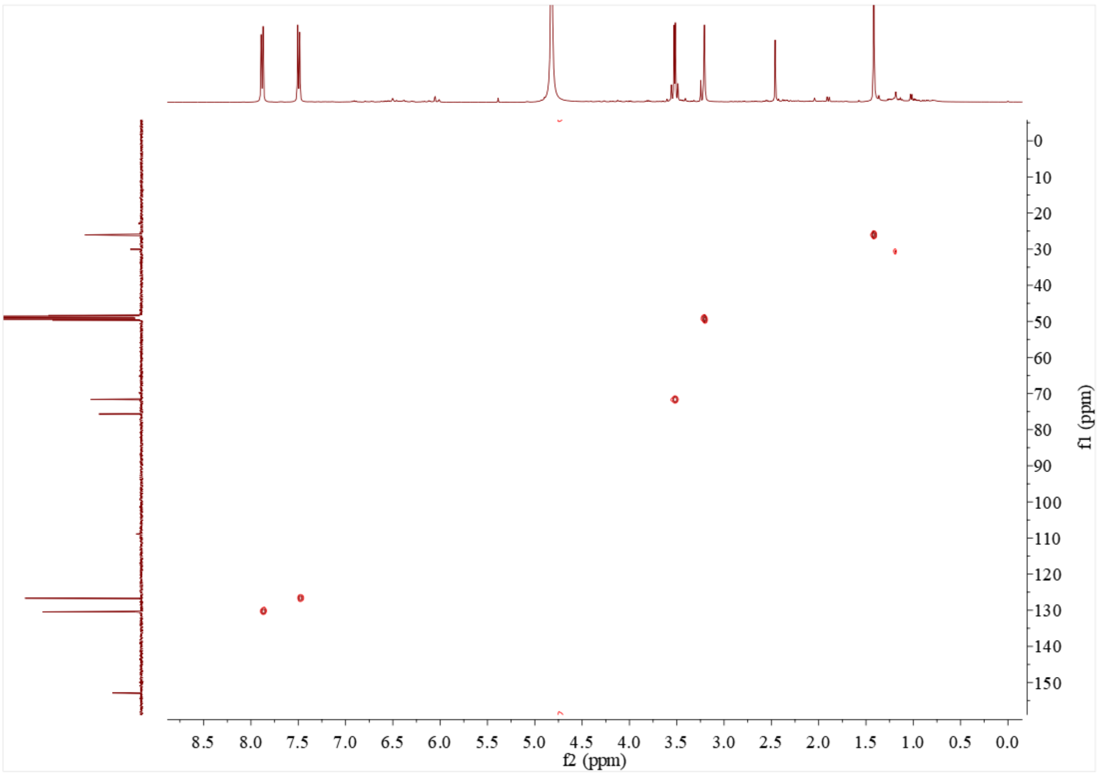

Figure S20. The HSQC spectrum of compound **3** in CD_3OD.

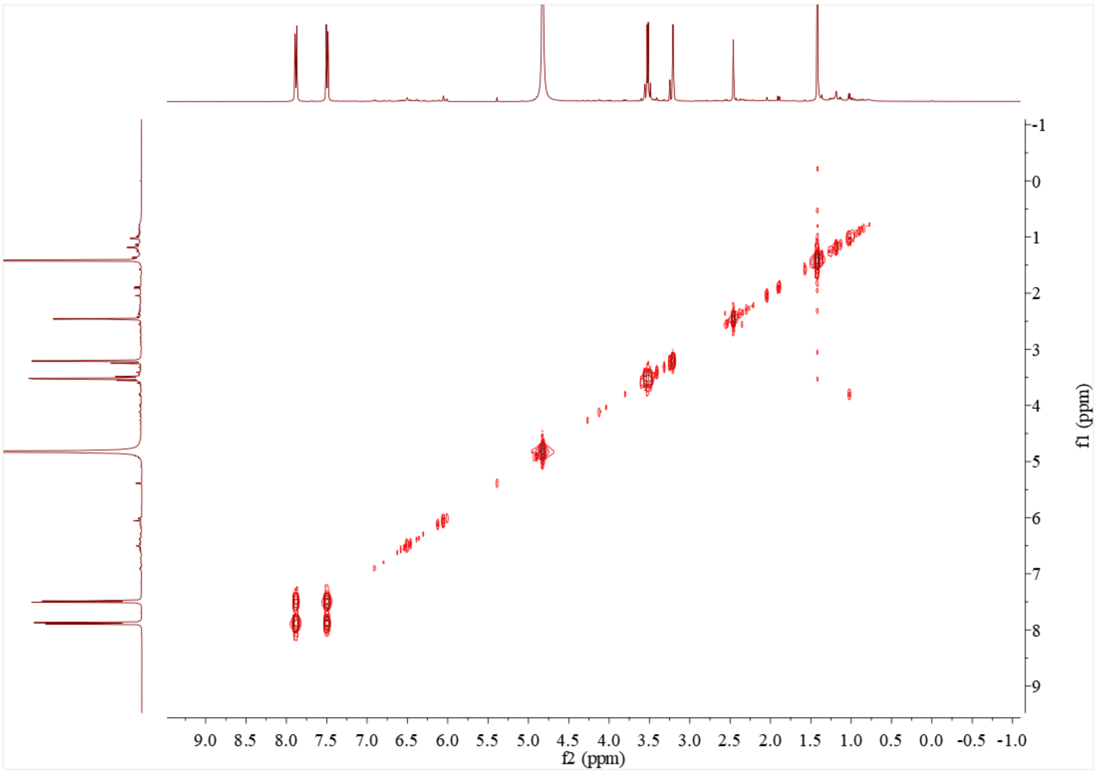

Figure S21. The ^{1}H-^{1}H COSY spectrum of compound **3** in CD_3OD.

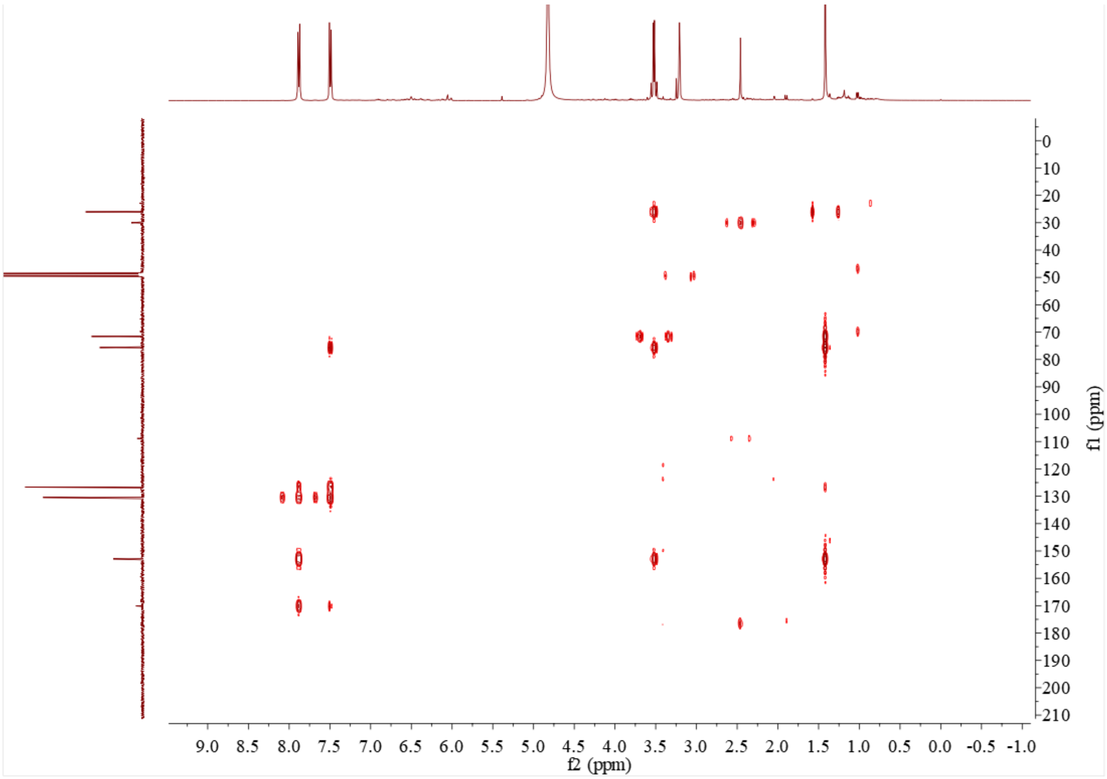

Figure S22. The HMBC spectrum of compound **3** in CD_3OD.

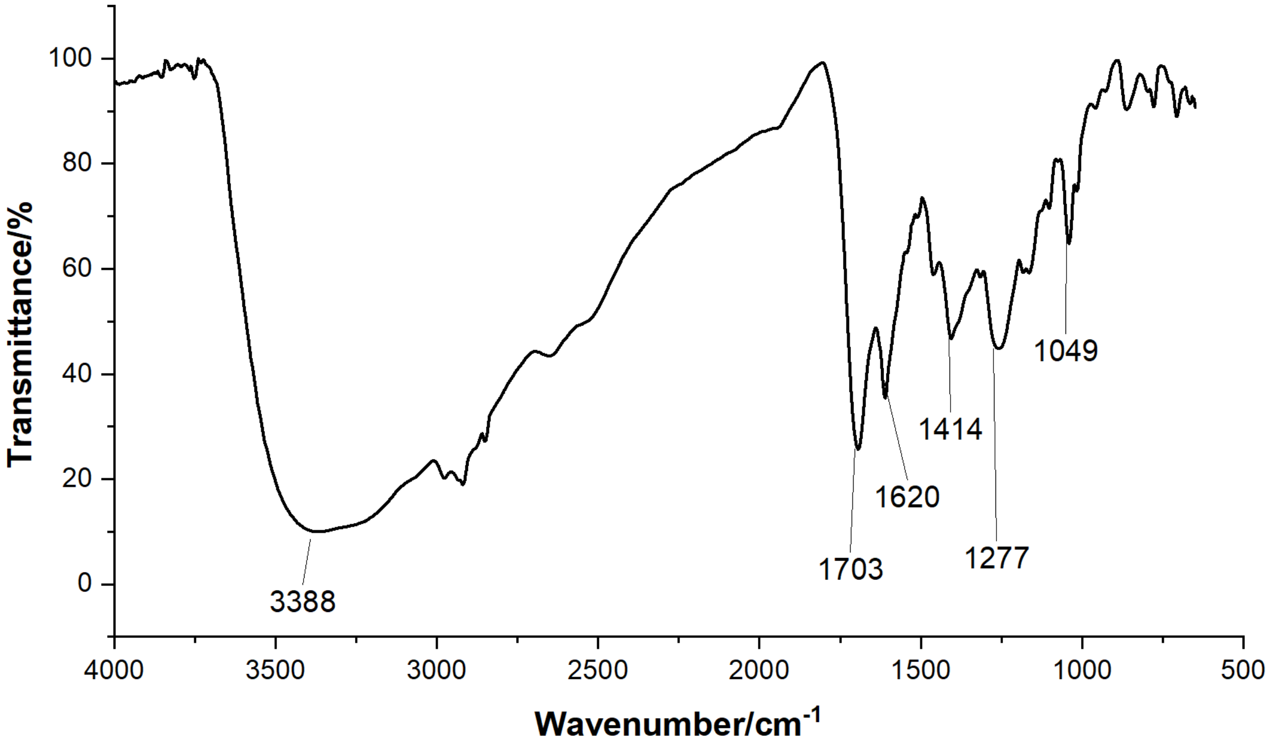

Figure S23. IR spectrum of compound **3**.

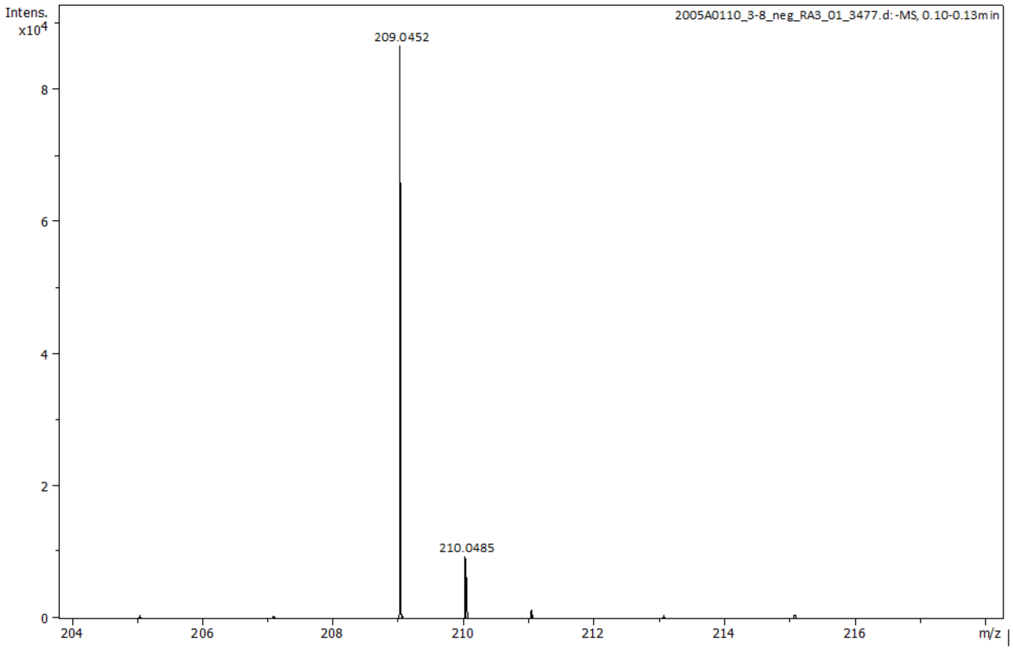

Meas. m/z	Ion Formula	m/z	z	err [ppm]	err [mDa]	mSigma	rdb	e⁻ Conf	N-Rule
209.0452	C10H9O5	209.0455	1-	1.5	0.3	2.3	6	even	ok

Figure S24. The HRESIMS spectrum of compound **6**.

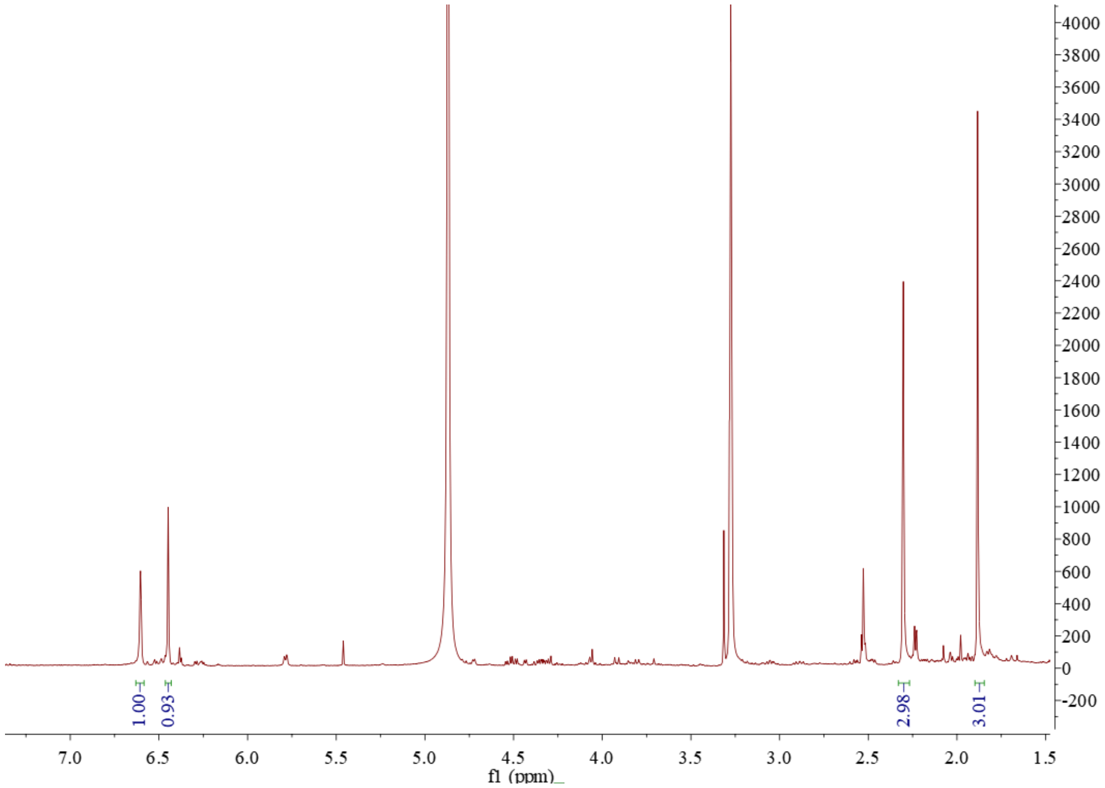

Figure S25. The ^{1}H NMR (400MHz) spectrum of compound **6** in CD_3OD.

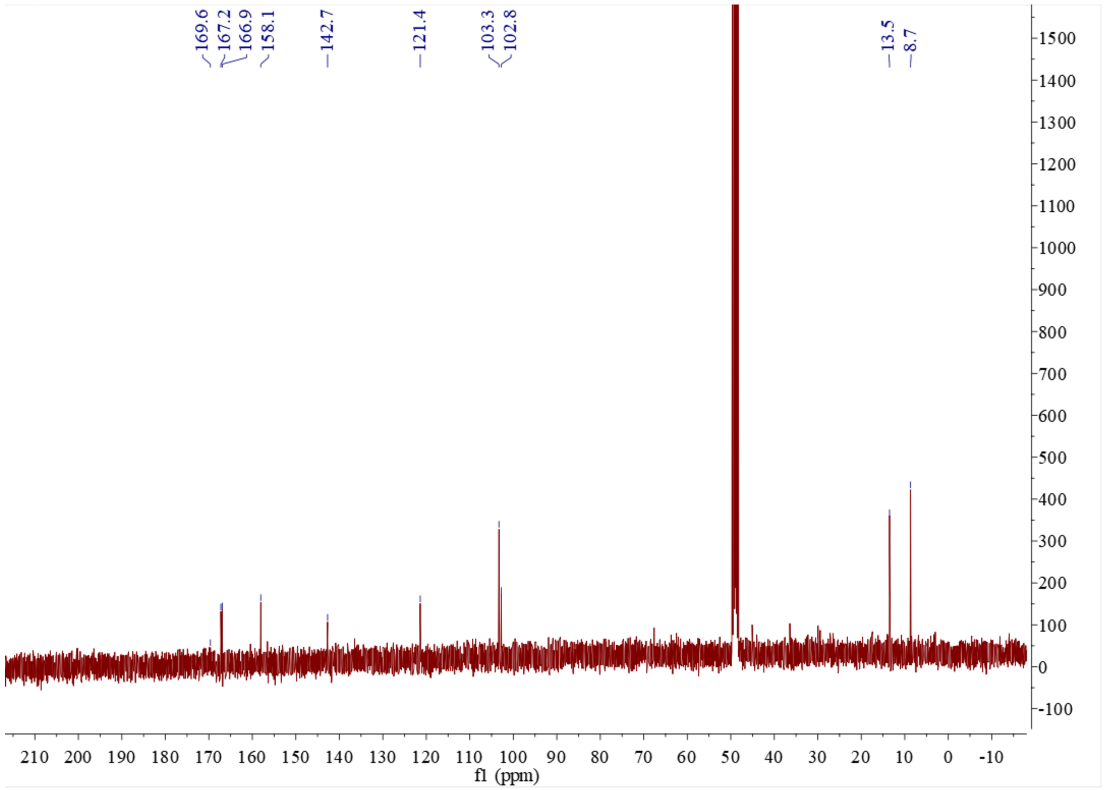

Figure S26. The ^{13}C NMR (100MHz) spectrum of compound **6** in CD_3OD.

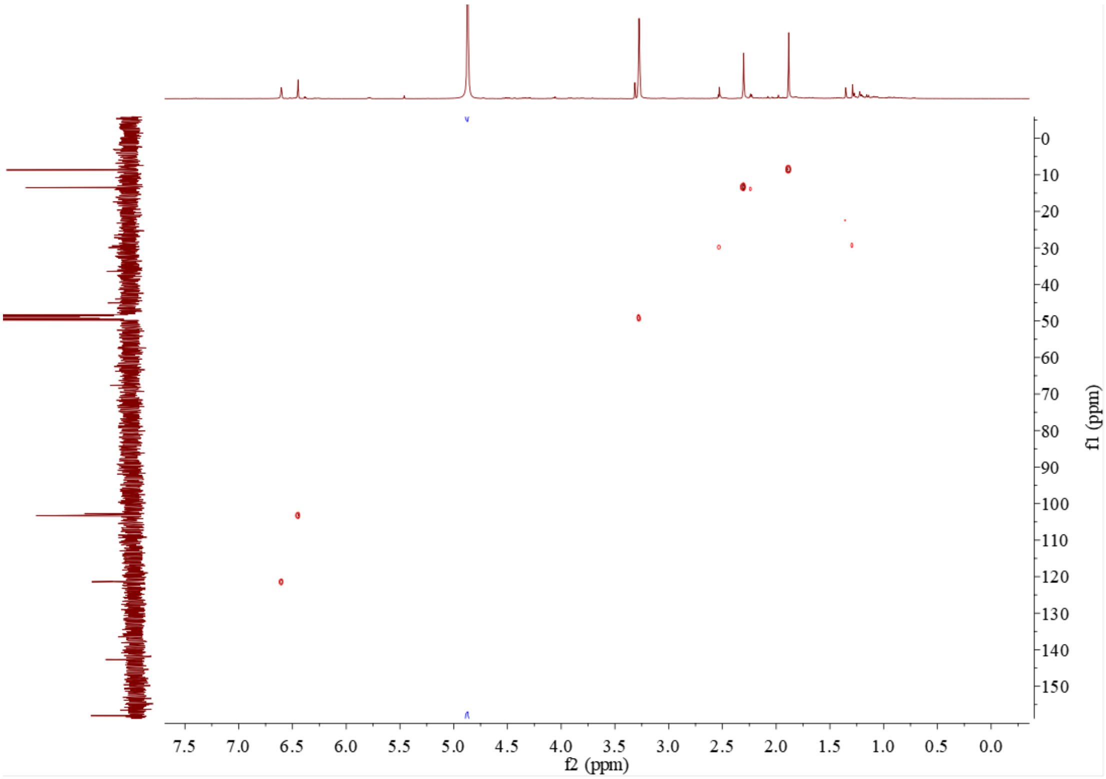

Figure S27. The HSQC spectrum of compound **6** in CD_3OD.

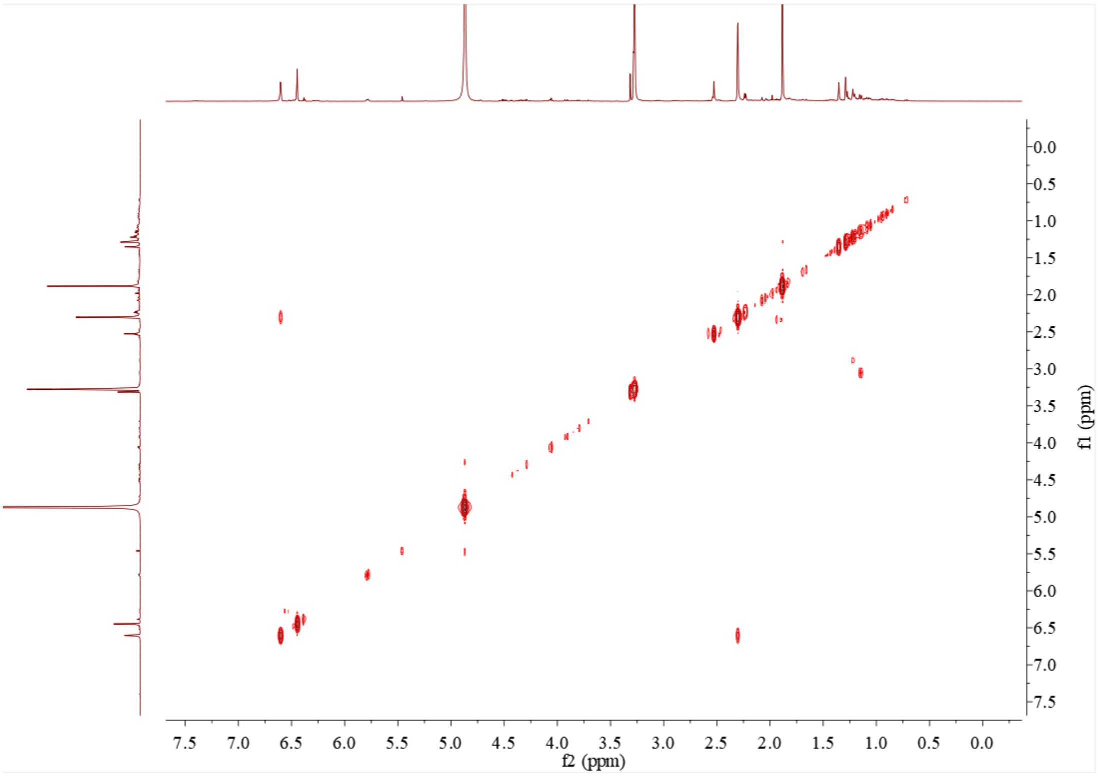

Figure S28. The ^1H-^1H COSY spectrum of compound **6** in CD_3OD.

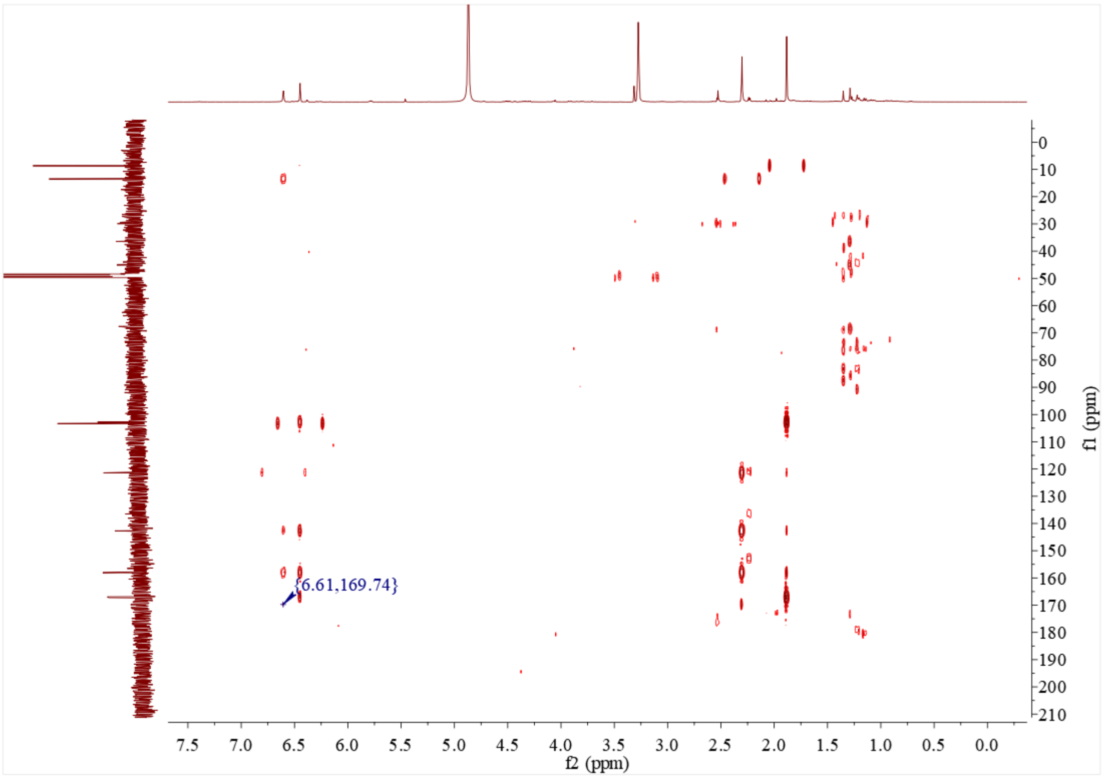

Figure S29. The HMBC spectrum of compound **6** in CD_3OD.

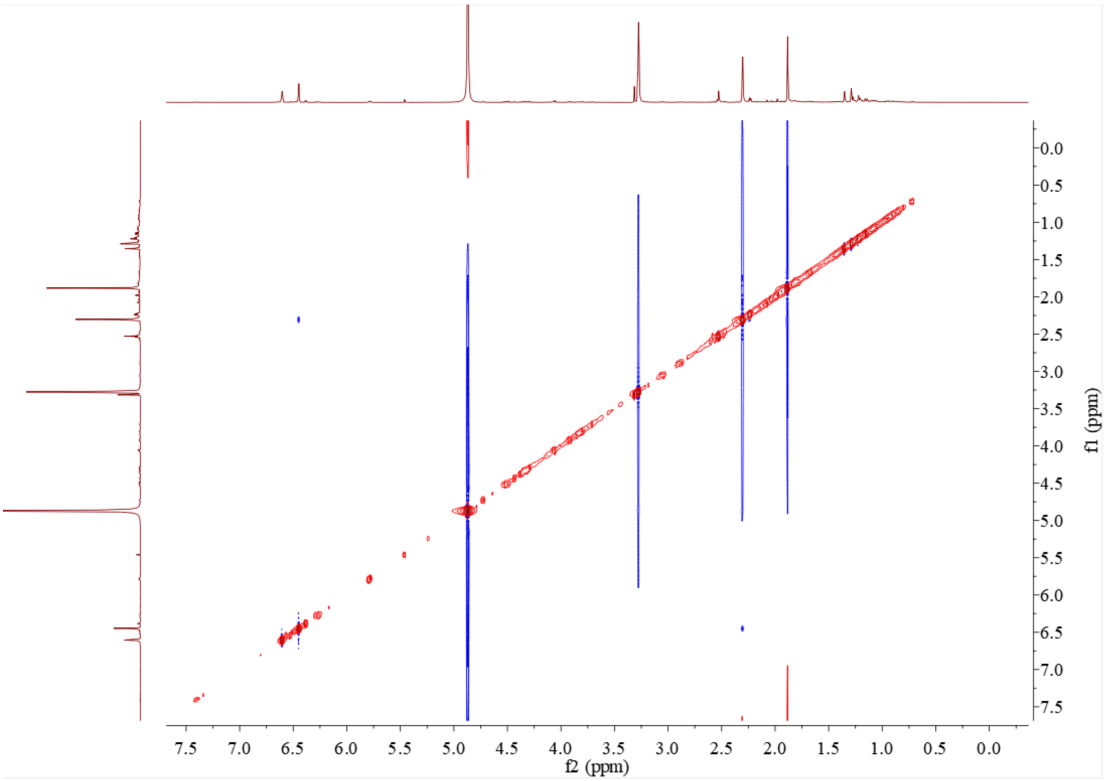

Figure S30. The NOESY spectrum of compound **6** in CD_3OD.

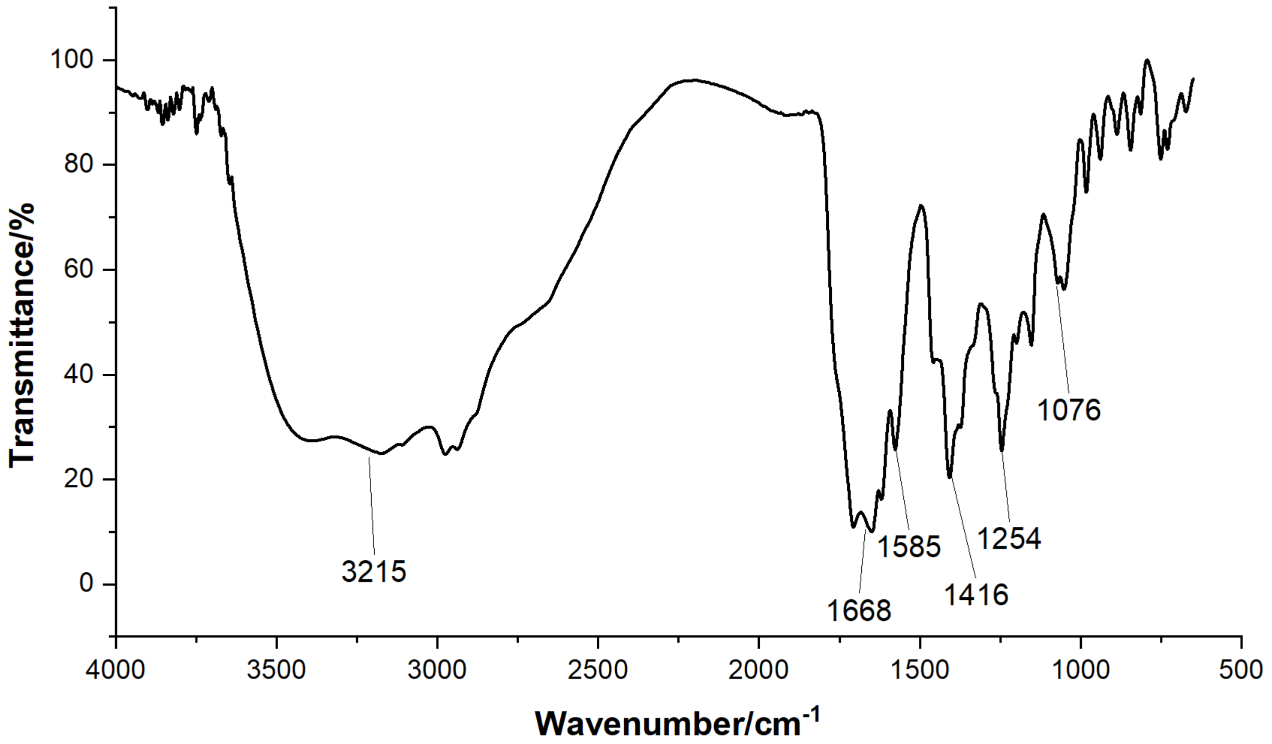

Figure S31. IR spectrum of compound **6.**

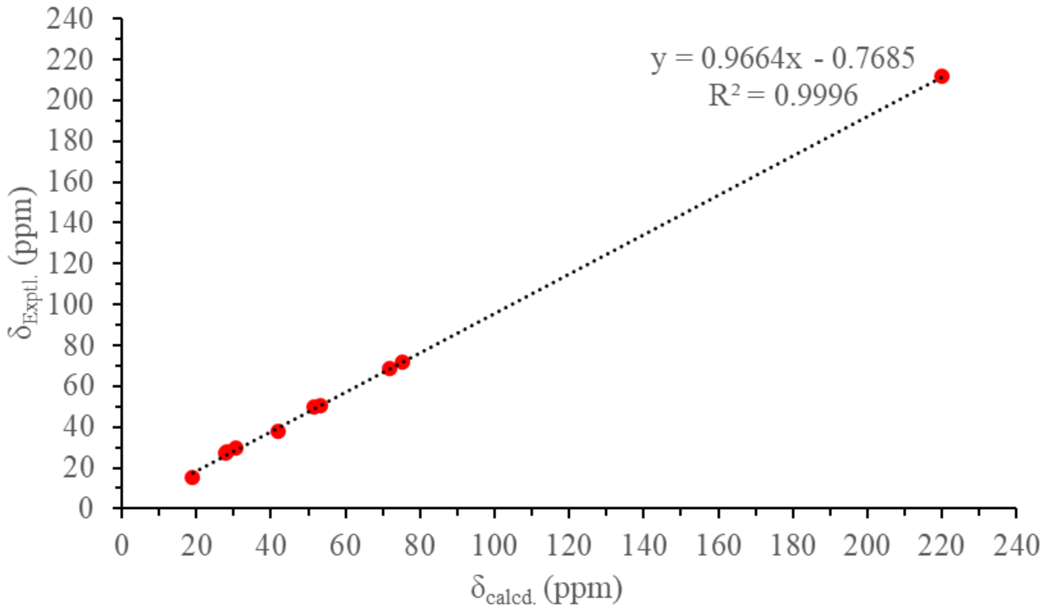

Figure S32. Linear correlation between the experimental ^{13}C NMR chemical shifts for **2**.

Functional		Solvent?		Basis Set		Type of Data	
mPW1PW91		PCM		6-311+G(d,p)		Unscaled Shifts	
		**2**	**(3*S*,4*R*,6*S*)**	**(3*R*,4*R*,6*S*)**			
		DP4+	100.00%	0.00%	-	-	-
Nuclei	sp2?	xperimenta	Isomer 1	Isomer 2	Isomer 3	Isomer 4	Isomer 5
C		71.9	75.4	77.3			
C		38.1	41.8	42.8			
C		50.3	53.3	51.0			
C	x	211.9	220.0	222.3			
C		49.6	51.7	52.3			
C		15.6	18.9	20.0			
C		68.5	71.7	71.8			
C		28	28.2	28.0			
C		27.2	27.9	27.8			
C		29.8	30.5	26.4			
H		2.24	2.12	2.13			
H		2.44	2.41	2.29			
H		2.01	1.99	1.95			
H		1.31	1.19	0.75			
H		1.52	1.47	1.47			
H		1.38	1.33	1.35			
H		1.28	1.25	1.29			
H		1.41	1.40	1.37			

Functional	Solvent?		Basis Set		Type of Data	
mPW1PW91	PCM		6-311+G(d,p)		Unscaled Shifts	
	**(3*S*,4*R*,6*S*)**	**(3*R*,4*R*,6*S*)**				
	Isomer 1	Isomer 2	Isomer 3	Isomer 4	Isomer 5	Isomer 6
sDP4+ (H data)	99.48%	0.52%	-	-	-	-
sDP4+ (C data)	99.90%	0.10%	-	-	-	-
sDP4+ (all data)	100.00%	0.00%	-	-	-	-
uDP4+ (H data)	97.43%	2.57%	-	-	-	-
uDP4+ (C data)	99.85%	0.15%	-	-	-	-
uDP4+ (all data)	100.00%	0.00%	-	-	-	-
DP4+ (H data)	99.99%	0.01%	-	-	-	-
DP4+ (C data)	100.00%	0.00%	-	-	-	-
DP4+ (all data)	100.00%	0.00%	-	-	-	-

Figure S33. The DP4 probability analysis

Table S1. Energy of all conformers of compound **2**.

compound	conformer	E_{tot} (a.u.)	$G_{298.15}$ (a.u.)	P (%)
	**2-a**	-616.360310	-616.155547	15.83%
	**2-b**	-616.360558	-616.154885	7.85%
	**2-c**	-616.360549	-616.155133	10.21%
	**2-d**	-616.360254	-616.154269	4.09%
**2**	**2-e**	-616.360769	-616.154774	6.98%
	**2-f**	-616.360756	-616.154634	6.01%
	**2-g**	-616.359980	-616.154986	8.73%
	**2-h**	-616.361079	-616.154589	5.73%
	**2-i**	-616.360969	-616.154774	6.98%
	**2-j**	-616.361144	-616.154300	4.22%

**2-k**	-616.360508	-616.154345	4.43%
**2-l**	-616.360631	-616.154215	3.86%
**2-m**	-616.361432	-616.154372	4.56%
**2-n**	-616.358216	-616.152092	0.41%
**2-o**	-616.361160	-616.154549	5.50%
**2-p**	-616.360881	-616.153879	2.70%
**2-q**	-616.357909	-616.151947	0.35%
**2-r**	-616.358380	-616.152224	0.47%
**2-s**	-616.358406	-616.151934	0.34%
**2-t**	-616.358148	-616.151908	0.33%
**2-u**	-616.358650	-616.152150	0.43%

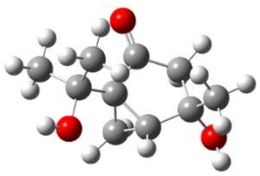

2-a

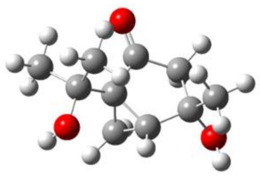

2-b

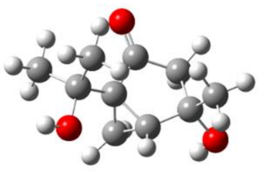

2-c

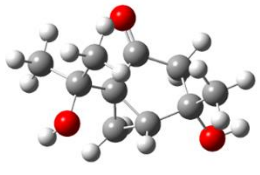

2-d

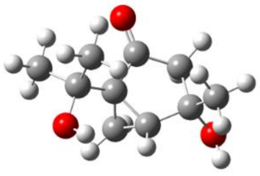

2-e

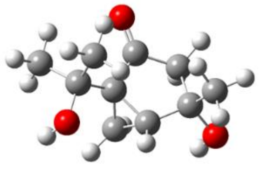

2-f

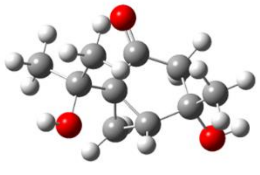

2-g

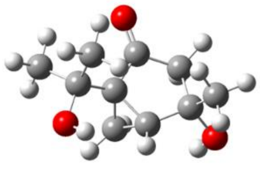

2-h

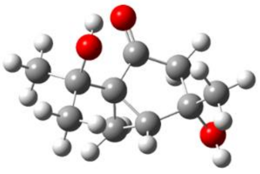

2-i

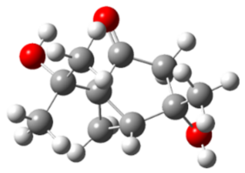

2-j

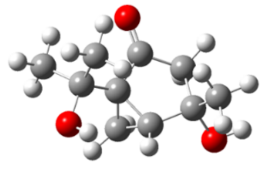

2-k

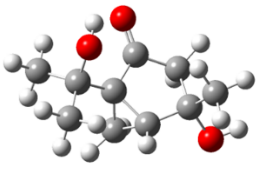

2-l

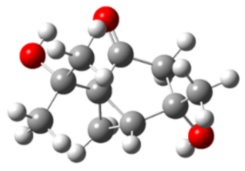

2-m

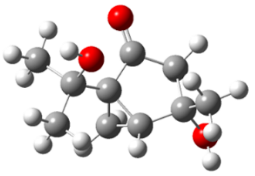

2-n

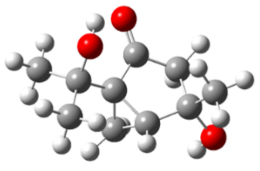

2-o

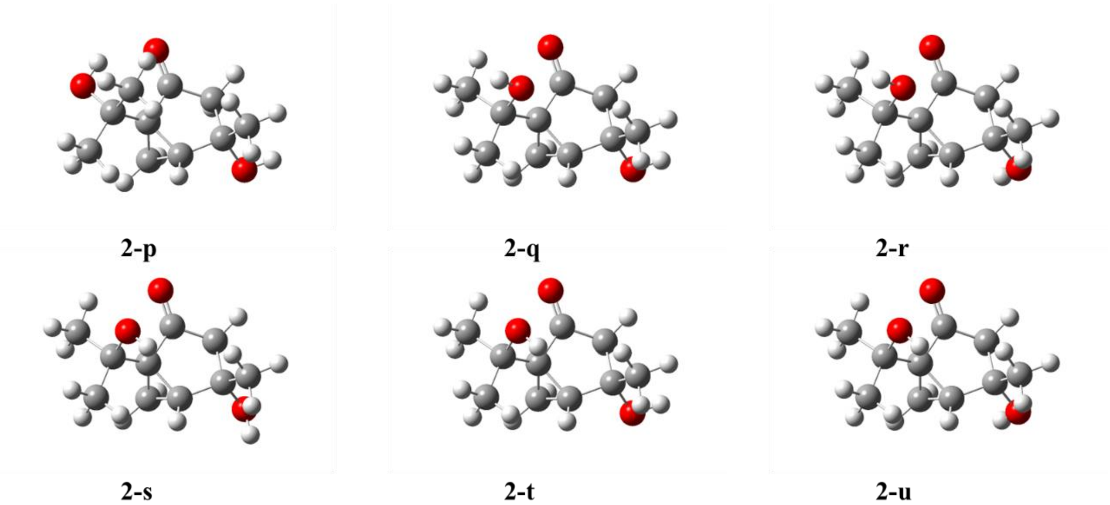

Figure S34. wB97XD/TZVP optimized low-energy conformers of compound **2**.

Table S2. Cartesian coordinates of the low-energy reoptimized conformers of **2** calculated at PBE1PBE/TZVP level of theory in IEFPCM for methanol.

**2-a**			
Atom	X axis (Å)	Y axis (Å)	Z axis (Å)
C	1.983325	-0.202841	0.129111
C	0.712761	-0.993501	-0.170863
C	-0.448581	-0.036523	-0.35212
C	0.142548	1.332078	-0.274881
C	1.656571	1.221294	-0.333658
C	0.113002	-0.832763	-1.5199
C	-1.853893	-0.309656	0.165435
C	-2.894901	0.375767	-0.715882
C	-1.978774	0.129374	1.623071
O	-2.020288	-1.729915	0.086743
O	-0.463091	2.371691	-0.143474
O	3.105423	-0.66383	-0.617215
C	2.28015	-0.24754	1.623856
H	0.546283	-1.92092	0.361243
H	1.984282	1.37116	-1.365169
H	2.118566	1.995343	0.277105
H	0.665674	-0.315048	-2.293312
H	-0.531873	-1.628133	-1.865187
H	-2.80255	0.023371	-1.744412
H	-3.901557	0.146876	-0.356848
H	-2.759585	1.456703	-0.698114
H	-1.232483	-0.383083	2.232716
H	-2.970943	-0.121927	2.005715

H	-1.840701	1.205779	1.722607
H	-2.907687	-1.937471	0.388714
H	3.324807	-1.548886	-0.314394
H	3.167724	0.344703	1.850814
H	2.451146	-1.276623	1.949244
H	1.43931	0.150469	2.195838

**2-b**			
Atom	X axis (Å)	Y axis (Å)	Z axis (Å)
C	1.979176	-0.203392	0.128202
C	0.708069	-0.991311	-0.174359
C	-0.452246	-0.036626	-0.362576
C	0.144888	1.332624	-0.293945
C	1.658876	1.217985	-0.346914
C	0.111693	-0.836619	-1.525714
C	-1.856133	-0.311139	0.181916
C	-2.908823	0.421626	-0.642408
C	-1.923106	0.078416	1.652883
O	-2.11204	-1.718177	0.148547
O	-0.457958	2.374283	-0.168012
O	3.105026	-0.673308	-0.606285
C	2.263662	-0.237395	1.62566
H	0.53638	-1.91781	0.358464
H	1.990065	1.358308	-1.37872
H	2.121565	1.995676	0.258672
H	0.662928	-0.321637	-2.301769
H	-0.510244	-1.646924	-1.880605
H	-2.858128	0.110576	-1.689563
H	-3.904578	0.196848	-0.256068
H	-2.744234	1.497286	-0.59934
H	-1.191072	-0.494301	2.225244
H	-2.917267	-0.134505	2.047917
H	-1.717612	1.140637	1.781581
H	-2.425614	-1.954371	-0.727528
H	3.322357	-1.554901	-0.291999
H	3.151789	0.353065	1.854996
H	2.427437	-1.26436	1.961182
H	1.419675	0.169017	2.187062

**2-c**			
Atom	X axis (Å)	Y axis (Å)	Z axis (Å)
C	1.983214	-0.208751	0.146588
C	0.712637	-0.999767	-0.138531
C	-0.443792	-0.046502	-0.352577

C	0.152292	1.32301	-0.315864
C	1.6656	1.203588	-0.38437
C	0.114481	-0.876579	-1.49724
C	-1.849765	-0.301026	0.172676
C	-2.889122	0.363289	-0.726411
C	-1.971871	0.177086	1.618027
O	-2.019725	-1.722297	0.131381
O	-0.451077	2.365411	-0.198993
O	3.130587	-0.789251	-0.459731
C	2.258424	-0.176723	1.64081
H	0.544469	-1.915581	0.41159
H	1.98261	1.314755	-1.424943
H	2.13897	2.000106	0.18753
H	0.650518	-0.375263	-2.29384
H	-0.527844	-1.684397	-1.81687
H	-2.801853	-0.021441	-1.743719
H	-3.895744	0.150975	-0.35731
H	-2.74816	1.443525	-0.742399
H	-1.226679	-0.321262	2.240576
H	-2.964383	-0.061332	2.007746
H	-1.830327	1.25541	1.688447
H	-2.909662	-1.919579	0.432736
H	2.93887	-0.939862	-1.389292
H	3.163242	0.397466	1.845112
H	2.389447	-1.18969	2.024106
H	1.425654	0.2895	2.170636

**2-d**			
Atom	X axis (Å)	Y axis (Å)	Z axis (Å)
C	1.972476	-0.21882	0.129147
C	0.70393	-0.99842	-0.170048
C	-0.451434	-0.039802	-0.366893
C	0.151013	1.327917	-0.314582
C	1.664174	1.203081	-0.377896
C	0.106179	-0.854519	-1.522835
C	-1.854116	-0.303528	0.185774
C	-2.906922	0.428907	-0.638658
C	-1.91247	0.095649	1.654563
O	-2.117458	-1.709434	0.162789
O	-0.446787	2.3724	-0.188382
O	3.049671	-0.833498	-0.568857
C	2.23958	-0.213588	1.63038
H	0.529932	-1.919832	0.369539
H	1.98711	1.317974	-1.415488

H	2.135963	1.991193	0.207708
H	0.654462	-0.349813	-2.30714
H	-0.519371	-1.667191	-1.865508
H	-2.862598	0.110351	-1.683856
H	-3.901883	0.211502	-0.246153
H	-2.737228	1.504037	-0.603776
H	-1.181979	-0.478528	2.227413
H	-2.906319	-0.108332	2.055054
H	-1.699478	1.157263	1.776102
H	-2.438037	-1.949395	-0.709715
H	3.863327	-0.386246	-0.322265
H	3.14346	0.356881	1.856766
H	2.367399	-1.233563	1.995926
H	1.407369	0.246695	2.167289

**2-e**			
Atom	X axis (Å)	Y axis (Å)	Z axis (Å)
C	1.986141	-0.200658	0.122012
C	0.716053	-0.986275	-0.194237
C	-0.450637	-0.028588	-0.346787
C	0.140228	1.339728	-0.243919
C	1.654304	1.234639	-0.299765
C	0.106389	-0.795519	-1.534269
C	-1.866171	-0.317553	0.159092
C	-2.895468	0.343692	-0.745697
C	-2.014791	0.136655	1.608251
O	-2.12459	-1.721916	0.072981
O	-0.469208	2.375581	-0.101831
O	3.104942	-0.637682	-0.642664
C	2.289727	-0.288704	1.613445
H	0.574372	-1.93518	0.308467
H	1.983711	1.414613	-1.325918
H	2.112721	1.992239	0.333957
H	0.653924	-0.262764	-2.301091
H	-0.539972	-1.586132	-1.889622
H	-2.808012	-0.050834	-1.759365
H	-3.901401	0.139428	-0.375521
H	-2.73778	1.421064	-0.773201
H	-1.273827	-0.359047	2.241851
H	-3.011008	-0.116445	1.97412
H	-1.873647	1.213271	1.696151
H	-1.6916	-2.158389	0.810834
H	3.335839	-1.526034	-0.358778
H	3.176197	0.299758	1.853876

H	2.466437	-1.326609	1.906438
H	1.450251	0.088691	2.201156

2-f			
Atom	X axis (Å)	Y axis (Å)	Z axis (Å)
C	1.978987	-0.209928	0.145619
C	0.707698	-0.997141	-0.144484
C	-0.447525	-0.045525	-0.362931
C	0.154993	1.324274	-0.331611
C	1.668319	1.200812	-0.392961
C	0.11314	-0.876338	-1.505059
C	-1.852316	-0.30241	0.188181
C	-2.902565	0.417954	-0.649768
C	-1.915085	0.116146	1.65136
O	-2.113419	-1.708834	0.182313
O	-0.445031	2.368876	-0.219736
O	3.129067	-0.795857	-0.449783
C	2.242509	-0.172507	1.641828
H	0.534045	-1.913379	0.404037
H	1.989725	1.305996	-1.432862
H	2.141832	1.998756	0.176831
H	0.648575	-0.376351	-2.302646
H	-0.506529	-1.698266	-1.835551
H	-2.854873	0.084298	-1.690115
H	-3.898615	0.205773	-0.257186
H	-2.733321	1.493508	-0.62988
H	-1.18597	-0.450145	2.233752
H	-2.909984	-0.083482	2.051387
H	-1.703094	1.179391	1.759915
H	-2.442437	-1.958503	-0.684292
H	2.945255	-0.94719	-1.380812
H	3.146827	0.400744	1.850844
H	2.368397	-1.184126	2.03036
H	1.406301	0.297473	2.162825

2-g			
Atom	X axis (Å)	Y axis (Å)	Z axis (Å)
C	1.975205	-0.218658	0.129613
C	0.708127	-1.000658	-0.171654
C	-0.44785	-0.039032	-0.36043
C	0.149033	1.327827	-0.295784
C	1.662099	1.206329	-0.36592
C	0.107623	-0.848086	-1.522237
C	-1.850767	-0.302652	0.168015

C	-2.894676	0.393913	-0.700983
C	-1.957576	0.132729	1.628241
O	-2.029634	-1.721105	0.086563
O	-0.451131	2.369975	-0.159635
O	3.052447	-0.825003	-0.57587
C	2.247118	-0.223689	1.630018
H	0.538191	-1.92382	0.365518
H	1.981338	1.329912	-1.403603
H	2.134156	1.990742	0.224407
H	0.657496	-0.339172	-2.302921
H	-0.540567	-1.645117	-1.857093
H	-2.813588	0.045786	-1.731937
H	-3.899492	0.169549	-0.334115
H	-2.752038	1.473793	-0.679245
H	-1.210044	-0.388416	2.228938
H	-2.948207	-0.110593	2.019922
H	-1.808881	1.207594	1.729763
H	-2.911511	-1.923447	0.407656
H	3.865417	-0.377486	-0.327507
H	3.149111	0.3491	1.858221
H	2.380653	-1.245962	1.987024
H	1.414495	0.228467	2.17325

2-h

Atom	X axis (Å)	Y axis (Å)	Z axis (Å)
C	1.986594	-0.208047	0.138351
C	0.71583	-0.991864	-0.166159
C	-0.4459	-0.036014	-0.346239
C	0.150137	1.333792	-0.277753
C	1.663815	1.220321	-0.342799
C	0.108087	-0.830894	-1.515911
C	-1.862701	-0.310024	0.165062
C	-2.889281	0.336039	-0.753623
C	-2.010429	0.172978	1.604735
O	-2.124636	-1.715084	0.106578
O	-0.456496	2.372669	-0.148795
O	3.130445	-0.766895	-0.493788
C	2.271068	-0.228048	1.631087
H	0.572483	-1.932549	0.350003
H	1.983295	1.369296	-1.377845
H	2.13305	1.997781	0.2581
H	0.639045	-0.309757	-2.303198
H	-0.535326	-1.632469	-1.850799
H	-2.803045	-0.079755	-1.758847

H	-3.895832	0.142556	-0.379492
H	-2.728237	1.412114	-0.803397
H	-1.269594	-0.31043	2.247843
H	-3.006638	-0.072935	1.975441
H	-1.869541	1.251023	1.672248
H	-1.700219	-2.137489	0.857537
H	2.938229	-0.876488	-1.429009
H	3.175298	0.341723	1.849588
H	2.408688	-1.253662	1.976622
H	1.440523	0.216086	2.18274

2-i

Atom	X axis (Å)	Y axis (Å)	Z axis (Å)
C	2.001687	-0.199929	0.118461
C	0.736636	-1.015766	-0.142673
C	-0.443949	-0.074765	-0.332196
C	0.143731	1.293916	-0.275609
C	1.651583	1.204573	-0.389096
C	0.116201	-0.894898	-1.483315
C	-1.86961	-0.281043	0.184637
C	-2.145321	-1.730799	0.553353
C	-2.872929	0.208006	-0.861973
O	-2.038122	0.453981	1.400669
O	-0.478144	2.317816	-0.080578
O	3.119605	-0.673011	-0.622963
C	2.309659	-0.188011	1.611304
H	0.606055	-1.936201	0.411542
H	1.940223	1.312823	-1.437448
H	2.129288	2.004662	0.173505
H	0.647689	-0.391237	-2.280274
H	-0.511957	-1.711079	-1.814034
H	-1.492838	-2.050608	1.366242
H	-3.179738	-1.824378	0.885759
H	-2.003397	-2.398513	-0.296818
H	-2.69785	1.257921	-1.101332
H	-3.88765	0.10086	-0.475214
H	-2.786964	-0.372902	-1.781596
H	-1.883951	1.382115	1.190277
H	3.374476	-1.530127	-0.271291
H	3.18946	0.425032	1.810765
H	2.498345	-1.202404	1.970916
H	1.465969	0.216047	2.174624

2-j

Atom	X axis (Å)	Y axis (Å)	Z axis (Å)
C	1.974581	-0.211452	0.150054
C	0.717821	-1.015389	-0.182196
C	-0.448588	-0.072233	-0.387734
C	0.135511	1.299045	-0.318946
C	1.646903	1.211923	-0.320013
C	0.145278	-0.865176	-1.543488
C	-1.863405	-0.274622	0.153563
C	-1.866772	-0.003581	1.661392
C	-2.409567	-1.666521	-0.123079
O	-2.747422	0.632625	-0.510537
O	-0.503375	2.328017	-0.233772
O	3.116893	-0.659711	-0.569566
C	2.231966	-0.255823	1.651937
H	0.561583	-1.947594	0.347173
H	2.011685	1.371289	-1.337626
H	2.075743	1.98884	0.310982
H	0.708709	-0.340547	-2.304407
H	-0.464418	-1.675589	-1.916227
H	-1.515559	1.006157	1.880432
H	-2.879991	-0.10631	2.052221
H	-1.220201	-0.713525	2.180936
H	-2.531827	-1.831576	-1.193438
H	-3.386844	-1.768044	0.350126
H	-1.754686	-2.438502	0.283408
H	-2.352169	1.510499	-0.428208
H	3.353009	-1.533911	-0.248313
H	3.106745	0.345473	1.902931
H	2.405051	-1.283546	1.980327
H	1.372019	0.132408	2.201741

**2-k**			
Atom	X axis (Å)	Y axis (Å)	Z axis (Å)
C	1.978983	-0.216176	0.122276
C	0.711696	-0.993053	-0.191485
C	-0.449794	-0.030917	-0.352311
C	0.146629	1.33609	-0.264745
C	1.660034	1.220583	-0.332604
C	0.101233	-0.811999	-1.533432
C	-1.86387	-0.310399	0.162809
C	-2.895074	0.353889	-0.737476
C	-2.000806	0.148834	1.611555
O	-2.130978	-1.713419	0.082697
O	-0.457443	2.374739	-0.120676

O	3.050894	-0.802228	-0.607692
C	2.262927	-0.263022	1.619722
H	0.567404	-1.937484	0.31758
H	1.980281	1.374536	-1.365928
H	2.128491	1.989569	0.280516
H	0.646093	-0.288659	-2.308086
H	-0.548454	-1.604572	-1.87789
H	-2.815416	-0.043391	-1.750725
H	-3.899672	0.1551	-0.360802
H	-2.73259	1.430439	-0.768306
H	-1.25918	-0.349884	2.241984
H	-2.99629	-0.097025	1.984303
H	-1.852617	1.22482	1.695824
H	-1.697679	-2.150276	0.820151
H	3.865549	-0.360829	-0.353819
H	3.165066	0.306067	1.856181
H	2.403426	-1.294799	1.945355
H	1.43404	0.170666	2.183165

**2-l**			
Atom	X axis (Å)	Y axis (Å)	Z axis (Å)
C	1.995102	-0.21486	0.12029
C	0.732858	-1.022076	-0.137755
C	-0.443022	-0.077728	-0.336907
C	0.14952	1.289607	-0.296159
C	1.656324	1.19041	-0.418196
C	0.11148	-0.912629	-1.479958
C	-1.867177	-0.273652	0.187782
C	-2.149582	-1.719339	0.567338
C	-2.872656	0.21366	-0.857663
O	-2.026278	0.470325	1.399601
O	-0.467295	2.316352	-0.099732
O	3.066512	-0.832316	-0.581056
C	2.286113	-0.165397	1.616092
H	0.600217	-1.93722	0.423484
H	1.93879	1.274389	-1.470408
H	2.140764	1.999661	0.126217
H	0.640385	-0.419298	-2.284543
H	-0.519365	-1.731469	-1.798642
H	-1.493502	-2.038939	1.377389
H	-3.182131	-1.803439	0.908048
H	-2.018851	-2.393194	-0.279767
H	-2.694473	1.261559	-1.103501
H	-3.886219	0.112681	-0.466252

H	-2.792654	-0.3728	-1.774327
H	-1.865861	1.395873	1.182317
H	3.880619	-0.376688	-0.351794
H	3.182245	0.427626	1.812714
H	2.439544	-1.173177	2.004722
H	1.453216	0.291574	2.154654

**2-m**			
Atom	X axis (Å)	Y axis (Å)	Z axis (Å)
C	1.974141	-0.217431	0.167853
C	0.718177	-1.021713	-0.152985
C	-0.443592	-0.082163	-0.387529
C	0.145942	1.289853	-0.356195
C	1.657313	1.194957	-0.368317
C	0.146818	-0.905095	-1.523061
C	-1.859101	-0.266902	0.158782
C	-1.860147	0.037318	1.660144
C	-2.411143	-1.662138	-0.087718
O	-2.739654	0.628793	-0.524913
O	-0.490254	2.321091	-0.284011
O	3.142001	-0.784446	-0.406937
C	2.206158	-0.186836	1.669358
H	0.560929	-1.944312	0.391529
H	2.010721	1.317465	-1.395807
H	2.098758	1.994262	0.224782
H	0.693037	-0.394573	-2.306822
H	-0.459244	-1.72853	-1.871975
H	-1.504296	1.050016	1.856925
H	-2.873674	-0.052127	2.053256
H	-1.216832	-0.663932	2.195163
H	-2.538288	-1.847422	-1.15422
H	-3.386949	-1.750479	0.390922
H	-1.757886	-2.428587	0.331588
H	-2.344545	1.507889	-0.456647
H	2.983638	-0.92791	-1.343893
H	3.097055	0.398745	1.900138
H	2.340564	-1.199253	2.052708
H	1.352878	0.26614	2.177254

**2-n**			
Atom	X axis (Å)	Y axis (Å)	Z axis (Å)
C	1.989389	-0.222392	0.120297
C	0.719746	-0.979931	-0.254559
C	-0.447475	-0.015744	-0.347657

C	0.151274	1.351266	-0.179228
C	1.666961	1.236878	-0.209793
C	0.115887	-0.701414	-1.582299
C	-1.851703	-0.298657	0.164279
C	-2.220223	-1.781093	0.1042
C	-2.884165	0.51953	-0.612638
O	-1.821187	0.108496	1.539768
O	-0.44492	2.39142	-0.03045
O	3.11227	-0.615217	-0.663632
C	2.279779	-0.411167	1.604775
H	0.57521	-1.957516	0.186151
H	2.017704	1.48665	-1.214177
H	2.113196	1.948937	0.482955
H	0.663979	-0.107713	-2.302684
H	-0.503793	-1.471352	-2.019725
H	-1.523812	-2.385564	0.685097
H	-3.216341	-1.9128	0.532146
H	-2.254059	-2.156904	-0.918957
H	-2.667623	1.582635	-0.530564
H	-3.885332	0.327216	-0.218416
H	-2.876488	0.235969	-1.666665
H	-2.715734	0.05346	1.885206
H	3.334882	-1.522011	-0.437102
H	3.162255	0.161823	1.892868
H	2.457724	-1.466229	1.82725
H	1.432107	-0.077849	2.206863

**2-o**			
Atom	X axis (Å)	Y axis (Å)	Z axis (Å)
C	2.00147	-0.206436	0.135529
C	0.736773	-1.020542	-0.116365
C	-0.43925	-0.082782	-0.333483
C	0.153244	1.286549	-0.309772
C	1.660239	1.188263	-0.430684
C	0.11821	-0.92967	-1.465325
C	-1.865111	-0.273119	0.189326
C	-2.149071	-1.715413	0.580438
C	-2.867897	0.205597	-0.862565
O	-2.025222	0.481288	1.394161
O	-0.465314	2.313998	-0.124456
O	3.149022	-0.789289	-0.46359
C	2.285891	-0.129031	1.6263
H	0.604728	-1.932046	0.450959
H	1.940191	1.265103	-1.48496

H	2.146251	2.005111	0.099806
H	0.634341	-0.438359	-2.280771
H	-0.505973	-1.756694	-1.775361
H	-1.494463	-2.029333	1.393889
H	-3.182076	-1.795508	0.920619
H	-2.018494	-2.396301	-0.261066
H	-2.68952	1.251482	-1.116533
H	-3.882289	0.107461	-0.472634
H	-2.785781	-0.388439	-1.774161
H	-1.867191	1.405314	1.168945
H	2.958343	-0.958218	-1.390255
H	3.18167	0.466147	1.808286
H	2.437742	-1.129135	2.034627
H	1.44764	0.334952	2.149289

**2-p**			
Atom	X axis (Å)	Y axis (Å)	Z axis (Å)
C	1.96821	-0.225975	0.150854
C	0.714193	-1.021295	-0.177884
C	-0.447764	-0.074875	-0.390943
C	0.140875	1.295222	-0.337911
C	1.652322	1.199355	-0.348106
C	0.140504	-0.880963	-1.540679
C	-1.861172	-0.268055	0.15729
C	-1.857749	0.014581	1.66304
C	-2.414132	-1.659803	-0.10659
O	-2.744381	0.637509	-0.510056
O	-0.494264	2.326549	-0.25379
O	3.06141	-0.820739	-0.536761
C	2.210393	-0.233747	1.656203
H	0.556451	-1.949368	0.357178
H	2.010163	1.336428	-1.371463
H	2.08897	1.986329	0.265728
H	0.701058	-0.365024	-2.309201
H	-0.471688	-1.694284	-1.902828
H	-1.502295	1.02491	1.872684
H	-2.869834	-0.081545	2.058553
H	-1.211674	-0.693546	2.185753
H	-2.541775	-1.832028	-1.175236
H	-3.389794	-1.75357	0.371576
H	-1.760866	-2.43169	0.302796
H	-2.345739	1.514513	-0.435385
H	3.867946	-0.373766	-0.267178
H	3.101505	0.347988	1.903496

H	2.34857	-1.255908	2.011715
H	1.362805	0.207335	2.185023

2-q			
Atom	X axis (Å)	Y axis (Å)	Z axis (Å)
C	1.983642	-0.237186	0.118751
C	0.716513	-0.984817	-0.255533
C	-0.446571	-0.016515	-0.350631
C	0.155333	1.350026	-0.190174
C	1.670821	1.228939	-0.227126
C	0.111567	-0.708365	-1.584105
C	-1.849922	-0.293453	0.166908
C	-2.222139	-1.775308	0.117044
C	-2.882698	0.522122	-0.612276
O	-1.814838	0.122119	1.539788
O	-0.438196	2.391455	-0.040449
O	3.055656	-0.7694	-0.653192
C	2.262274	-0.402826	1.608564
H	0.570838	-1.961318	0.185567
H	2.016575	1.466932	-1.236095
H	2.122183	1.946669	0.457103
H	0.656961	-0.118414	-2.309051
H	-0.510659	-1.479523	-2.015618
H	-1.526476	-2.377628	0.700969
H	-3.21803	-1.901851	0.547065
H	-2.258305	-2.157959	-0.903481
H	-2.663341	1.585219	-0.538346
H	-3.883016	0.33516	-0.213398
H	-2.879407	0.231054	-1.664267
H	-2.708228	0.069189	1.888483
H	3.872261	-0.35751	-0.359478
H	3.157552	0.15402	1.895614
H	2.412465	-1.456646	1.848017
H	1.424731	-0.026472	2.199221

2-r			
Atom	X axis (Å)	Y axis (Å)	Z axis (Å)
C	1.990203	-0.230576	0.134324
C	0.72097	-0.985633	-0.236473
C	-0.442699	-0.021835	-0.348699
C	0.1591	1.347768	-0.201209
C	1.674603	1.229277	-0.238252
C	0.118198	-0.725493	-1.573409
C	-1.847992	-0.293129	0.167589

C	-2.220425	-1.775286	0.130821
C	-2.878194	0.515477	-0.622161
O	-1.815022	0.135797	1.536045
O	-0.436764	2.38865	-0.059046
O	3.138244	-0.742967	-0.530796
C	2.261672	-0.369769	1.623118
H	0.576201	-1.959892	0.209924
H	2.016766	1.462232	-1.250461
H	2.12881	1.956352	0.433296
H	0.649728	-0.13561	-2.310348
H	-0.497641	-1.505935	-1.997023
H	-1.525235	-2.37284	0.720197
H	-3.216366	-1.897571	0.561876
H	-2.25703	-2.167037	-0.886237
H	-2.659145	1.579193	-0.556959
H	-3.879617	0.331862	-0.224594
H	-2.871797	0.214903	-1.671467
H	-2.709184	0.087911	1.883504
H	2.954665	-0.776936	-1.473444
H	3.157907	0.188993	1.896612
H	2.408047	-1.419359	1.882278
H	1.419617	0.017076	2.199338

	**2-s**		
Atom	X axis (Å)	Y axis (Å)	Z axis (Å)
C	1.987025	-0.221773	0.112103
C	0.713267	-0.97566	-0.258241
C	-0.455313	-0.008063	-0.350255
C	0.148085	1.355854	-0.171243
C	1.663798	1.239958	-0.207049
C	0.111964	-0.690095	-1.58525
C	-1.864321	-0.294764	0.166497
C	-2.234418	-1.775114	0.076586
C	-2.894576	0.540575	-0.583901
O	-1.938841	0.11328	1.539012
O	-0.445625	2.395069	-0.007224
O	3.101069	-0.61224	-0.685248
C	2.295254	-0.419285	1.591728
H	0.56987	-1.956443	0.176612
H	2.012242	1.496715	-1.2104
H	2.112362	1.946868	0.489433
H	0.665609	-0.0957	-2.30079
H	-0.508098	-1.456228	-2.028313
H	-1.541991	-2.398691	0.645651

H	-3.230917	-1.907595	0.498934
H	-2.255862	-2.132329	-0.953163
H	-2.686011	1.601408	-0.462652
H	-3.893247	0.325606	-0.200245
H	-2.870013	0.295996	-1.646882
H	-1.37197	-0.467598	2.053352
H	3.324299	-1.520898	-0.466894
H	3.189867	0.140652	1.867534
H	2.461872	-1.477103	1.809398
H	1.46458	-0.073331	2.210257

2-t

Atom	X axis (Å)	Y axis (Å)	Z axis (Å)
C	1.981153	-0.236526	0.11073
C	0.709965	-0.980919	-0.257976
C	-0.454268	-0.009137	-0.353711
C	0.152552	1.35437	-0.183944
C	1.667975	1.231435	-0.227839
C	0.107687	-0.699691	-1.586361
C	-1.862358	-0.289047	0.169155
C	-2.23778	-1.768623	0.08878
C	-2.892327	0.545225	-0.582825
O	-1.931194	0.126675	1.539664
O	-0.438109	2.39488	-0.017857
O	3.044212	-0.769245	-0.672826
C	2.276496	-0.40726	1.596693
H	0.565046	-1.959732	0.17921
H	2.009898	1.473227	-1.237171
H	2.123055	1.945734	0.457455
H	0.658623	-0.110618	-2.307833
H	-0.515299	-1.467742	-2.021812
H	-1.545774	-2.391501	0.659085
H	-3.233369	-1.895206	0.515082
H	-2.264007	-2.13182	-0.938757
H	-2.68056	1.606108	-0.467951
H	-3.89048	0.335191	-0.195169
H	-2.871541	0.294784	-1.644524
H	-1.368192	-0.456272	2.055937
H	3.864234	-0.357012	-0.389239
H	3.1837	0.136375	1.871063
H	2.41466	-1.462983	1.834716
H	1.45615	-0.015987	2.201844

2-u

Atom	X axis (Å)	Y axis (Å)	Z axis (Å)
C	1.987738	-0.23018	0.125775
C	0.714721	-0.98173	-0.240673
C	-0.450144	-0.014524	-0.351951
C	0.156569	1.352147	-0.194657
C	1.672017	1.2317	-0.239474
C	0.11392	-0.715454	-1.576965
C	-1.860138	-0.288743	0.169905
C	-2.235119	-1.769069	0.104192
C	-2.887556	0.53769	-0.594152
O	-1.931632	0.142032	1.535239
O	-0.436556	2.392032	-0.035331
O	3.128927	-0.743474	-0.550044
C	2.27418	-0.374357	1.611285
H	0.570881	-1.958887	0.200652
H	2.010062	1.468241	-1.252162
H	2.13042	1.955216	0.433014
H	0.64982	-0.124848	-2.310209
H	-0.502555	-1.49265	-2.005125
H	-1.543897	-2.386235	0.681611
H	-3.231187	-1.891275	0.530596
H	-2.260579	-2.142807	-0.919603
H	-2.677138	1.599718	-0.487857
H	-3.887096	0.330209	-0.208785
H	-2.862148	0.277707	-1.653467
H	-1.374742	-0.438742	2.060551
H	2.936452	-0.778461	-1.490905
H	3.183072	0.169471	1.872511
H	2.406277	-1.425922	1.869896
H	1.449319	0.02966	2.200671
